# Integration of Nickel Adsorption Procedures into the Hydrometallurgical Processing of Laterite Ore: Comparative Performance of Two Chelating Ion-Exchange Resins with Insight into the Adsorption Mechanism

**DOI:** 10.3390/ma19173734

**Published:** 2026-09-02

**Authors:** Roza Shayakhmetova, Anar Mukhametzhanova, Almira Kuandykova, Ainur Kali, Petr Osipov, Akmaral Rakhym

**Affiliations:** 1 Laboratory of Rare Metals, RSE “National Center of Complex Processing of Mineral Raw Materials of the Republic of Kazakhstan”, Zhandossov St., 67, Almaty 050036, Kazakhstan; rozozhka@mail.ru (R.S.); a.kuandykova@satbayev.university (A.K.); osipovapi@mail.ru (P.O.); 2 Department of Physical Chemistry, Catalysis and Petrochemistry, Faculty of Chemistry and Chemical Technology, Farabi University, Al-Farabi Av., 71, Almaty 050040, Kazakhstan; a.rakhym@cmrp.kz; 3 Department of Metallurgy and Mineral Processing, Satbayev University, 22a Satpaev St., Almaty 050013, Kazakhstan; 4 Laboratory of Special Methods of Mineral Raw Materials Processing, RSE “National Center of Complex Processing of Mineral Raw Materials of the Republic of Kazakhstan”, Zhandossov St., 67, Almaty 050036, Kazakhstan

**Keywords:** nickel recovery, selective adsorption, laterite leach solution, metal separation, ion-exchange resin regeneration

## Abstract

Selective Ni(II) recovery from polymetallic sulfate leachates is challenging because of competition from accompanying metal ions, particularly Mg(II). This study systematically evaluated selective Ni(II) recovery using commercial chelating ion-exchange resins and assessed their adsorption performance and regeneration behavior in the context of their integration into a conceptual flowsheet for oxidized nickel ore processing. The ore was characterized by XRF, XRD, and SEM–EDS, while Seplite LSC 495 (bispicolylamine) and Seplite LSC 773 (iminodiacetate) were evaluated using selectivity tests, kinetic and equilibrium studies, FTIR spectroscopy, and adsorption–desorption experiments. Both resins exhibited high selectivity for Ni(II) over Co(II) and Mg(II). Under the optimized conditions, Seplite LSC 773 achieved 99.36 ± 0.08% Ni(II) recovery from the model solution and 98.59 ± 0.36% from the actual leach solution, whereas Seplite LSC 495 achieved 94.62 ± 0.21% and 94.23 ± 0.21%, respectively. The equilibrium data exhibited a Giles S-type profile without reaching a distinct saturation plateau within the investigated concentration range, and the Freundlich model provided the most appropriate description of the data, consistent with adsorption on energetically heterogeneous binding sites. FTIR spectral changes indicated the involvement of the chelating functional groups in Ni(II) binding. Among the regeneration conditions investigated, 20 wt.% H_2_SO_4_ provided the highest Ni(II) desorption efficiency; however, desorption remained limited to approximately 42%, indicating that regeneration remains a major constraint on practical application. The experimental findings were incorporated into a conceptual flowsheet integrating sulfuric acid leaching, selective Ni recovery, subsequent Co separation, and MgSO_4_·7H_2_O production. Further work should focus on optimizing regeneration, extending cyclic testing, evaluating continuous-flow operation, and validating the integrated process at the pilot scale.

## 1. Introduction

In recent years, increasing attention has been given to the processing of technogenic and natural ultramafic raw materials rich in magnesium and iron because of the need for the rational utilization of mineral resources and the incorporation of refractory materials into industrial processing strategies [1,2,3,4]. Such systems contain not only major components but also their associated valuable metals, including nickel and cobalt; the recovery of these metals requires an integrated technological approach [5,6,7]. Acid leaching results in the formation of magnesium-bearing solutions with complex chemical compositions, creating specific conditions for the subsequent stages of metal separation and selective recovery [8,9].

According to international statistics, nickel is considered a strategically important metal, and its demand has steadily increased in recent years because of the development of the battery industry [10] and the production of stainless steels [11] and specialty alloys [12,13]. Globally, most nickel is consumed within the metallurgical sector (more than 60–65%) [14]. However, the most rapidly growing segment is the production of lithium-ion batteries for electric vehicles [15,16], where nickel is used to increase the energy densities of cathode materials. In this context, two global trends have emerged: the expansion of the raw material base and the diversification of supply sources.

In Kazakhstan, the nickel industry is still in the early stages of development, and industrial production remains limited. However, to date, active geological exploration programs and preparations for the launch of new production facilities are underway according to the investment development strategy of Kazakhstan [17]. In particular, the development of cobalt–nickel deposits such as the Belogorskoye deposit (East Kazakhstan Region) requires the establishment of processing facilities that can meet an expected production capacity of approximately 13.5 thousand tons of nickel per year through the processing of up to 1.6 million tons of ore [17]. Thus, Kazakhstan is regarded as a promising participant in the global nickel market, with a strategic focus on integration into international supply chains for battery materials and high-technology industries [18].

To recover nickel from multicomponent polymetallic ores and their leaching products, various adsorbent materials, including ion exchangers, chelating resins, carbon-based sorbents, carbon nanomaterials, oxide and hydroxide sorbents, biosorbents [19], functionalized polymeric materials [20], composites, and clay- and zeolite-based adsorbents [21], have been employed. Their performance levels are typically evaluated in terms of Ni(II) selectivity, sorption capacity, kinetics, multicomponent matrix resistance, and regeneration ability.

Sorption directly from ore slurries makes it possible to avoid costly filtration and thickening processes [22,23]. In terms of both capital and operating expenditures, this procedure competes with solvent extraction and precipitation and may be considered an effective alternative for nickel ore processing [24,25,26,27,28,29,30,31]. Therefore, investigating the economic feasibility of processing low-grade nickel-bearing materials containing less than 1.0% Ni using existing technologies remains highly relevant. In this context, studies on low-grade sulfide ores are particularly important, as silicic acid and colloidal silica species can hinder sorption processes, whereas temperature optimization and pH control are critical factors for achieving industrially viable sorption rates [32,33].

For the selective recovery of nickel from polymetallic leach solutions, chelating ion-exchange resins containing iminodiacetic [34,35], bispicolylamine [35,36], aminophosphonic [37], and sulfonic acid [38] functional groups have been extensively investigated. These materials can selectively concentrate Ni(II) from acidic and weakly acidic media containing competing metal ions, including Fe, Al, Mg, Mn, Cu, Co, and Zn. For example, the bispicolylamine-functionalized resin Dowex M4195 exhibited high selectivity toward Ni and Co at pH 1–4 in the presence of Fe, Al, Mg, Mn, and Cu [35]. In a resin-in-pulp configuration, Ni and Co recoveries exceeded 99% after five counter-current stages, with Ni loading reaching 100 g kg^−1^ resin. The iminodiacetate resin Amberlite IRC 748R achieved a Ni capacity of 112.4 mg g^−1^ in column experiments and produced a 20–25-fold enrichment of Ni in the eluate, demonstrating its potential for processing lateritic HPAL solutions [34]. The bispicolylamine-functionalized Lewatit TP 220 was likewise effective for Ni(II) recovery from sulfate media, with adsorption being only weakly affected by pH within the range of 2–5 and reaching equilibrium within 72 h; H_2_SO_4_ solutions at concentrations ≥ 5% enabled effective Ni desorption and resin reuse [36]. Nearly complete Ni recovery from weakly acidic sulfate solutions, followed by 85–95% desorption of the loaded Ni, has also been reported for synthetic ion-exchange sorbents of the CYBBER series [38]. For low-grade sulfide Ni–Cu ores, direct application of the styrene–divinylbenzene-based chelating resin Purolite S930 in pulp enabled >99% recovery of Cu, Ni, and Co at pH 2.7–3.0, demonstrating the applicability of ion exchange to poorly filterable magnesium–silicate systems [39].

Despite these advances, an important gap remains between the performance demonstrated under simplified conditions and that required for complex hydrometallurgical streams. A substantial proportion of previous adsorption studies have relied on model solutions [35,36], whereas actual polymetallic ore leachates contain multiple competing ions [30], high dissolved-solid concentrations, and interfering impurities [39]. Such solution complexity can substantially affect metal selectivity, resin loading, and regeneration behavior. Therefore, evaluating chelating resins under conditions representative of real leach solutions, while considering both selective metal separation and subsequent resin regeneration, is essential for assessing their practical applicability.

Accordingly, this study aimed to assess the feasibility of selective Ni(II) recovery from complex polymetallic sulfate leach solutions using two commercial chelating ion-exchange resins, Seplite LSC 495 and Seplite LSC 773, within the context of oxidized nickel ore processing. Particular attention was given to the influence of operating conditions and competing metal ions on Ni(II) separation, as well as to the equilibrium and thermodynamic characteristics of the adsorption process. The study further examined the involvement of resin functional groups in Ni(II) binding and evaluated the regeneration and short-term reuse of the sorbents. Finally, the experimental findings were used to develop a conceptual hydrometallurgical flowsheet linking selective ion exchange with the sequential recovery of valuable components from the leach solution. Thus, the work bridges fundamental adsorption assessment with the practical requirements and current limitations of integrating selective ion exchange into complex nickel ore processing.

## 2. Materials and Methods

### 2.1. Materials and Reagents

The leach solution was obtained by sulfuric acid leaching of oxidized magnesium–iron–nickel ores from the Belogorskoye deposit (East Kazakhstan Region, Kazakhstan).

Sulfuric acid (96 wt.% H_2_SO_4_, JSC “Base No. 1 of Chemical Reagents”, Staraya Kupavna, Moscow, Russia) was used as the leaching reagent. Sodium hydroxide (3 M NaOH, analytical grade, LLP “Labkhimprom”, Almaty, Kazakhstan) was employed as a neutralizing agent during the hydrolytic purification of pregnant leach solutions from iron.

To improve the filtration properties of iron-containing hydrated precipitates and promote the formation of a suitable cake structure, the flocculant Praestol^®^ 2500 (Ashland Inc., Wilmington, DE, USA) was added directly to the pulp prior to filtration without pretreatment.

Model adsorption solutions were prepared by dissolving nickel sulfate heptahydrate (NiSO_4_·7H_2_O), cobalt sulfate heptahydrate (CoSO_4_·7H_2_O), and magnesium sulfate heptahydrate (MgSO_4_·7H_2_O) supplied by Junsei Chemical Co., Ltd. (Tokyo, Japan).

The purified solution was subsequently subjected to nickel sorption using the macroporous chelating ion-exchange resins Seplite^®^ LSC 495 (bispicolylamine functional groups; Sunresin New Materials Co., Ltd., Xi’an High-Tech Industrial Development Zone, Xi’an, China) and Seplite^®^ LSC 773 (iminodiacetate functional groups; Sunresin New Materials Co., Ltd., Xi’an High-Tech Industrial Development Zone, Xi’an, China). All the experiments were performed using deionized water.

### 2.2. Determination of the Technological Parameters of the Leaching Process

The main technological parameters of the sulfuric acid leaching process for laterite ore were investigated. The process included ore preparation, acid leaching, solution purification, and subsequent nickel sorption and desorption stages.

During the first stage, the ore was crushed to a particle size fraction of −2 + 0.5 mm. The material was then subjected to leaching with 20 wt.% sulfuric acid at a temperature of 85–90 °C for 3 h using a solid-to-liquid ratio (S:L) of 1:4 and a stirring rate of 400 rpm. Under these conditions, nickel and magnesium extraction efficiencies of 84.0 ± 1.8% and 92.5 ± 0.5%, respectively, were achieved. After three-stage washing and filtration, a silica-rich residue (SiO_2_ cake) was separated. The resulting pregnant leach solution contained (g/L) 2.22 g of Ni, 9.6 g of Fe, and 34.5 g/L of Mg.

The pregnant leach solution was subsequently purified from iron by precipitation at pH values of 3.8–4.0, which was followed by three-stage washing and drying of the precipitate (Fe cake). After the iron removal stage, the composition of the solution changed significantly and contained 1.68 g/L of Ni, 0.33 g/L of Co, and 16.0 g/L of Mg.

Thus, the selected technological parameters ensured the formation of a multicomponent solution suitable for subsequent sorption studies.

### 2.3. Adsorption Experiments

Prior to use, the ion-exchange resins LSC 495 and LSC 773 were pretreated to remove residual organic impurities by repeatedly washing them with distilled water. Sorbent activation was performed under static conditions using a 0.5 M sulfuric acid (H_2_SO_4_) solution at a phase:volume ratio of 1:10, ensuring complete conversion of the resin into the H^+^ form. After activation, the excess acid was removed by washing with distilled water until a neutral pH was achieved [36].

A model solution with a metal ion content similar to that of the pregnant solution after ore was leached was used: 1.5 g/L of Ni^2+^, 0.3 g/L of Co^2+^, and 14 g/L of Mg^2+^. Adsorption experiments were conducted under static conditions for 72 h with continuous agitation using a SHO-2D incubator shaker (Daihan Science Green Park, Wonju-si, Republic of Korea) at a rotation speed of 120 rpm. The desired pH was maintained by adding H_2_SO_4_ and NaOH solutions. The effect of the S:L ratio was investigated over a range of 1:2 to 1:20.

For kinetic studies, aliquots were withdrawn at predetermined time intervals during adsorption. After each experiment, the phases were separated by vacuum filtration. Thermodynamic parameters were evaluated over a temperature range of 5–35 °C. To study isotherm modeling, adsorption was performed over a Ni^2+^ concentration range from 2 to 300 g/L until the maximum solubility of the salt was reached.

All experiments were performed in triplicate. The metal ion concentrations in the initial and equilibrium solutions were determined via atomic absorption spectrometry (AAS) using a Shimadzu AA-7000 instrument (Shimadzu Corporation, Kyoto, Japan).

The equilibrium adsorption capacity (q_e_) values of the resins were calculated using the following equation:

(1) $$q_{e}=\frac{C_{0}-C_{e}}{m}\cdot V$$

where q_e_ is the amount of metal ions adsorbed per unit mass of adsorbent (g g^−1^), C_0_ is the initial concentration of the metal ions in solution (g L^−1^), C_e_ is the equilibrium concentration of the metal ions remaining in solution (g L^−1^), V is the working volume of the solution (L), and m is the mass of the sorbent (g).

The recovery of each metal ion was calculated according to the following equation:

(2) $$R=\frac{C_{0}-C_{e}}{C_{0}}\cdot 100\%$$

where C_0_ is the initial concentration of the metal ion in solution (g L^−1^), and C_e_ is the equilibrium concentration of the metal ion remaining in solution (g L^−1^).

Throughout this study, Ni(II) recovery (%) refers to the fraction of Ni(II) removed from the liquid phase by adsorption, whereas adsorption capacity (q_t_ or q_e_) denotes the amount of Ni(II) adsorbed per unit mass of resin.

### 2.4. Desorption and Regeneration Experiments

Desorption experiments were conducted to evaluate the regeneration performance of the sorbents. Prior to desorption, the resins were loaded with metal ions under the optimized adsorption conditions: 4 g of sorbent (LSC 495 or LSC 773) was mixed with 20 mL of solution containing 1.5 g/L Ni^2+^, 0.3 g/L Co^2+^, and 14.0 g/L Mg^2+^ and agitated at 120 rpm for 5 h at room temperature. The pH during this preceding adsorption stage was adjusted to 1 for LSC 495 and 3 for LSC 773, according to the optimized conditions established for the respective resins. After determining the amount of adsorbed metal ions, the Ni-loaded sorbent was subjected to desorption.

Sulfuric acid was selected as the desorbing agent to maintain the sulfate medium established during the preceding ore-leaching process and to avoid introducing additional anionic species into the process stream. For desorption, 4 g of spent sorbent was mixed with a 10–30 wt.% H_2_SO_4_ solution at a phase ratio of 1:2 and stirred for 2 h at 120 rpm using a SHO-2D incubator shaker at room temperature. The initial pH of the desorption medium was not independently adjusted, since the H_2_SO_4_ concentration served as the controlled experimental variable. The solid phase was then separated by vacuum filtration, washed with distilled water, and dried at 60 °C for 12 h.

The concentrations of desorbed Ni^2+^, Co^2+^, and Mg^2+^ ions in the regeneration filtrate were determined via atomic absorption spectrometry (AAS). To assess the stability and reusability of the sorbents, three consecutive adsorption–desorption cycles were performed.

The desorption efficiency (D, mg/g) was calculated using the following equation:

(3) $$D=\frac{C\times V}{m}$$

where C is the concentration of the desorbed ion (mg L^−1^), V is the volume of the acid solution (L), and m is the mass of the adsorbent (g).

### 2.5. Sorbent Characterization

Microscopic studies were conducted using an OLYMPUS BX 51 (Olympus Corporation, Tokyo, Japan) optical microscope. The morphologies and elemental compositions of the samples were studied using a JEOL JSM-7600F scanning electron microscope (JEOL Ltd., Akishima, Tokyo, Japan) equipped with an Oxford INCA Energy 350 (Oxford Instruments, Abingdon, UK) energy-dispersive analysis system. The images were captured at an accelerating voltage of 15 kV and a working distance of 12.0 mm in high vacuum mode (HighVac) with a magnification of 300×.

X-ray fluorescence semiquantitative analysis was performed using an Axios 1 kW X-ray fluorescence wavelength-dispersive spectrometer (PANalytical B.V., Almelo, The Netherlands). The data obtained were processed and interpreted in SuperQ software (Omnian 37).

X-ray phase analysis was performed on a D8 Advance diffractometer (Bruker AXS GmbH, Karlsruhe, Germany) using Cu–Kα X-ray radiation under the following conditions: a tube voltage of 40 kV, a current of 40 mA, a two-theta range of 3–90°, and a step size of 0.02°. Diffraction patterns were processed, and interplanar distances were calculated in EVA software, rel. 2020, Version 5.2.0.5 (Bruker AXS GmbH, Karlsruhe, Germany). Phases were interpreted using the PDXL-2 Search/Match program (Rigaku Corporation, Akishima, Tokyo, Japan) and the PDF-2, rel. 2020 (ICDD) database.

The element contents in the productive solutions were determined via atomic absorption spectroscopy using a Shimadzu AA-7000 spectrometer (Shimadzu Corporation, Kyoto, Japan). Measurements were performed in a flame/graphite furnace (depending on the element) in accordance with the manufacturer’s guidelines. Calibration was performed using standard comparison samples.

The FTIR spectra were recorded using a Nicolet Apex FTIR spectrometer (Thermo Scientific, Madison, WI, USA) equipped with a Smart iTX attenuated total reflectance (ATR) accessory with a diamond crystal. The data were collected in the spectral range of 4000–400 cm^−1^ with a resolution of 4 cm^−1^ and 10 coadded scans per spectrum.

The pH was determined using a FiveEasy (pH/mV) pH meter by Mettler Toledo (Greifensee, Switzerland).

### 2.6. Theory and Calculations

#### 2.6.1. Adsorption Kinetic Modeling

Adsorption kinetics were analyzed using the pseudo-first-order (PFO), pseudo-second-order (PSO), Elovich, Weber–Morris, Dumwald–Wagner, and chemisorption-diffusion models to characterize the kinetic behavior of Ni(II) uptake and evaluate the possible contributions of adsorption and mass-transfer processes.

Pseudo-first-order [40] and pseudo-second-order [41] models were fitted directly to the untransformed experimental data using nonlinear least-squares regression. The nonlinear forms of the PFO and PSO equations are expressed as follows:

(4) $$q_{t}=q_{e}(1-e^{-k_{1}t})$$

(5) $$q_{t}=\frac{k_{2}q_{e}^{2}t}{1+k_{2}q_{e}t}$$

where k_1_ (min^−1^) and k_2_ (g mg ^−1^ min^−1^) are the pseudo-first-order and pseudo-second-order rate constants, respectively; q_t_ is the amount of adsorbed solute (mg g^−1^); and q_e_ is its value at equilibrium (mg g^−1^).

The Elovich model was additionally applied to describe adsorption kinetics on an energetically heterogeneous surface [42]. Rather than using its linearized form, the experimental data were fitted directly using the nonlinear Elovich equation:

(6) $$q_{t}=\frac{1}{\beta }\ln\left(1+\alpha \beta t\right)$$

where α is the initial rate of adsorption (mg g^−1^ min^−1^), and β is related to the extent of surface coverage and activation energy for chemisorption (g mg^−1^).

In this work, the results were analyzed using the intraparticle diffusion models of Weber and Morris and Dumwald–Wagner. Unlike the PFO, PSO, and Elovich equations, these models were evaluated using their conventional linearized forms.

The most widely applied intraparticle diffusion equation of the Weber and Morris [43] model is expressed as follows (Equation (7)):

(7) $$q_{t}=k_{i}t^{0.5}+C$$

where $k_{i}$ is the intraparticle diffusion rate constant (mg g^−1^ min^−0.5^) and the intercept C reflects the boundary layer effect or surface adsorption (mg g^−1^). If a plot of q_t_ against t^0.5^ yielded a straight line passing through the origin, then intraparticle diffusion would be considered the rate-determining step for the adsorption process. When the plot had two or more intersecting lines, then two or more stages controlled the adsorption process [44].

The Dumwald–Wagner model is applicable for different adsorption systems, and the equation can be expressed as follows (Equation (8)):

(8) $$\ln\left(1-\frac{q_{t}}{q_{e}}\right)=K_{DW}t$$

where $K_{DW}$ is the film diffusion rate (min^−1^). If the plot of ln(1 − qt/qe) vs. t presented a straight line and passed through the origin, then intraparticle diffusion was the rate-limiting step; otherwise, the rate was controlled by film diffusion.

The chemisorption diffusion model is an empirical diffusion–chemisorption kinetic model developed by Sutherland [45]. The following form (Equation (9)) was used in this work:

(9) $$\frac{1}{q_{t}}=\frac{1}{q_{e}}+\frac{1}{K_{CD}t^{0.5}}$$

where K_CD_ (mg g^−1^ min^−0.5^) is the rate of mass transfer of metal ions from the liquid phase to the adsorption site.

All adsorption experiments were performed in triplicate, and the experimental values used for kinetic modelling represent the means of the three replicate measurements. PFO, PSO, and Elovich parameters were estimated by nonlinear least-squares regression using the untransformed data, whereas the Weber–Morris, Dumwald–Wagner, and chemisorption-diffusion parameters were determined from linear regression of their corresponding transformed equations. Parameter uncertainty was evaluated using the standard errors (SE) obtained from the regression analysis, and 95% confidence intervals (CI) were calculated for the fitted parameters. Model performance was evaluated using the coefficient of determination (R^2^) and, for the nonlinear kinetic models, the root-mean-square error (RMSE).

#### 2.6.2. Adsorption Isotherm Modeling

To elucidate the equilibrium adsorption mechanism of Ni^2+^ onto Seplite LSC 495 and Seplite LSC 773, the experimental equilibrium data were analyzed using four nonlinear adsorption isotherm models, namely, the Freundlich [46], Toth [47], Sips [48], and Redlich–Peterson [49] equations. Model parameters were estimated by nonlinear least-squares regression using OriginPro 2021b (OriginLab Corporation, Northampton, MA, USA).

The Freundlich model assumes adsorption on an energetically heterogeneous surface with a nonuniform distribution of adsorption energies. The model is expressed as follows:

(10) $$q_{e}=K_{F}C_{e}^{1/n}$$

where $q_{e}$ (g g^−1^) is the equilibrium adsorption capacity, $C_{e}$ (g L^−1^) is the equilibrium concentration of Ni^2+^, $K_{F}$ [(g g^−1^)(L g^−1^)] is the Freundlich adsorption coefficient, and n (dimensionless) is the heterogeneity parameter.

The Toth model extends the Langmuir equation by accounting for energetic heterogeneity while retaining a finite adsorption capacity. The nonlinear form of the equation is expressed as follows:

(11) $$q_{e}=\frac{q_{m}K_{T}C_{e}}{{\left[1+{\left(K_{T}C_{e}\right)}^{t}\right]}^{1/t}}$$

where $q_{m}$ is the theoretical maximum adsorption capacity (g g^−1^), $K_{T}$ is the Toth equilibrium constant (L g^−1^), and t (dimensionless) is the heterogeneity parameter. For t=1, the equation reduces to the Langmuir model.

The Sips (Langmuir–Freundlich) model combines the Freundlich equation at low concentrations with the Langmuir equation at high concentrations as follows:

(12) $$q_{e}=\frac{q_{m}(K_{S}C_{e}{)}^{n}}{1+(K_{S}C_{e}{)}^{n}}$$

where $q_{m}$ is the maximum adsorption capacity (g g^−1^), $K_{S}$ is the Sips equilibrium constant (L g^−1^), and n is the surface heterogeneity (dimensionless). When n=1, the Sips model is identical to the Langmuir equation.

The Redlich–Peterson model is a three-parameter hybrid equation that combines features of both the Langmuir and Freundlich models:

(13) $$q_{e}=\frac{K_{R}C_{e}}{1+a_{R}C_{e}^{g}}$$

where $K_{R}$ (L g^−1^) and $a_{R}$ (L g^−1^)^g^ are empirical constants and g (dimensionless) is the model exponent satisfying 0<g<1. When g=1, the equation reduces to the Langmuir isotherm, whereas values of g<1 indicate deviation from ideal monolayer adsorption.

The quality of nonlinear fitting was evaluated using the coefficient of determination ($R^{2}$) and the root mean square error (RMSE).

Isotherm model fitting was performed using the mean experimental values obtained from the three replicate experiments. The fitted model parameters are reported together with their corresponding standard errors (SE) obtained from the regression analysis.

## 3. Results and Discussion

### 3.1. Raw Material Characterization

**Figure 1:**
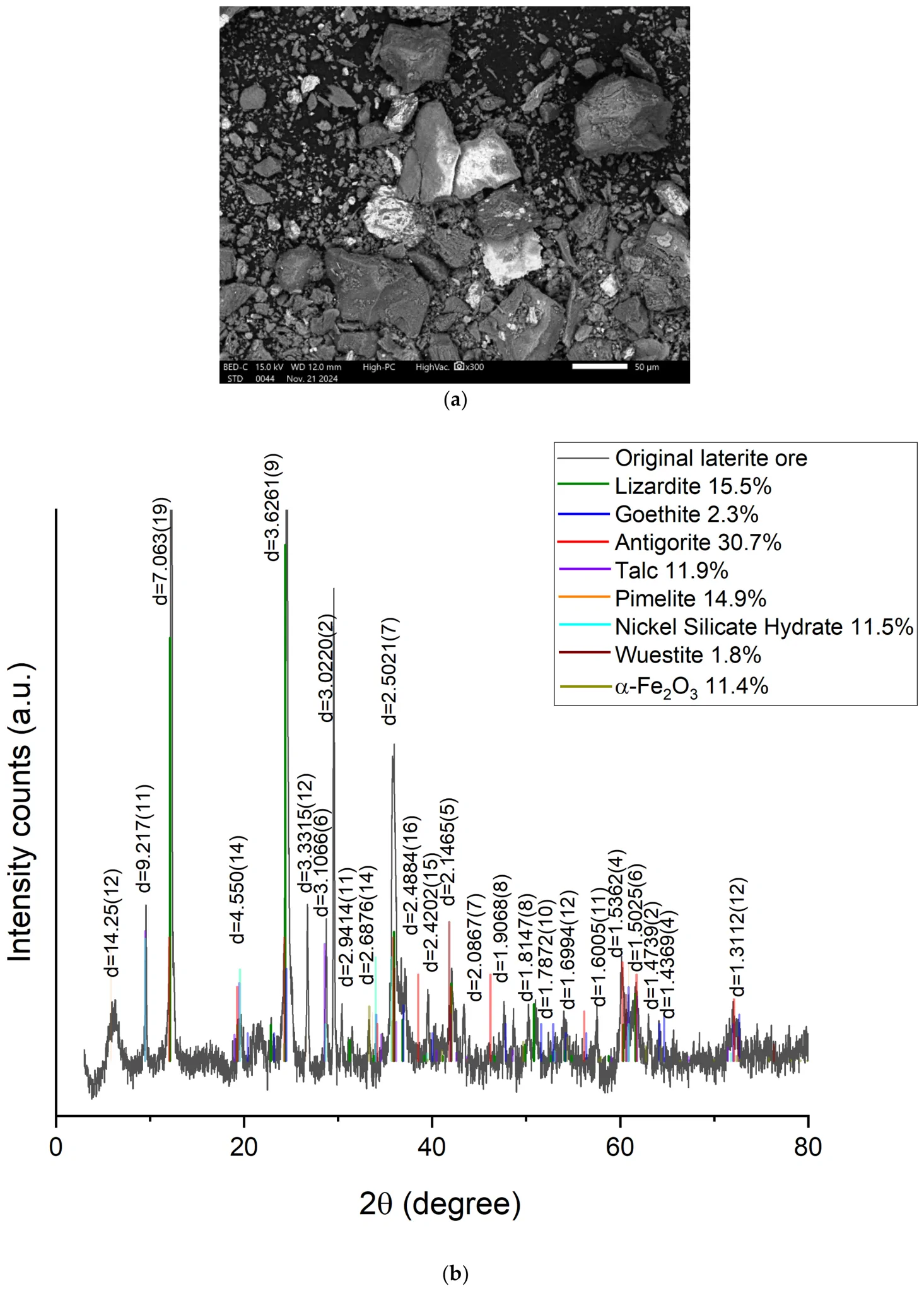
SEM micrograph (**a**) and X-ray diffraction pattern (**b**) of the initial ore sample.

The SEM and XRD results for oxidized magnesium–iron–nickel ores from the Belogorskoye deposit (East Kazakhstan Region) are shown in Figure 1.

X-ray diffraction analysis revealed that the raw ore has a complex polymineral composition and consists of iron-bearing, magnesium silicate, and nickel-bearing phases, including hematite (11.4%), goethite (2.3%), wüstite (1.8%), talc (11.9%), antigorite (30.7%), lizardite (15.5%), pimelite (14.9%), and hydrated nickel silicate (11.4%).

Iron is predominantly present in oxide and hydroxide minerals, namely, hematite (Fe_2_O_3_), goethite (FeOOH), and wüstite (FeO). Magnesium and silicon are concentrated mainly in silicate minerals of the serpentine group (antigorite and lizardite) and in talc. These minerals constitute the principal magnesium–silicate matrix of the ore. Nickel-bearing phases are represented by pimelite and hydrated nickel silicate, indicating that Ni is predominantly associated with silicate minerals. In addition, a portion of the nickel may occur as an isomorphic substitute for Mg and Fe within the crystal structure of serpentine minerals [3,9]. Such a phase composition suggests that nickel recovery during sulfuric acid leaching is largely governed by the extent of dissolution of the Ni-silicate and Mg–Fe silicate phases. The characteristic 2θ peak positions assigned to all crystalline phases identified in the original laterite ore are summarized in Table S3.

SEM analysis of the raw ore from the Belogorskoye deposit revealed a heterogeneous polydisperse structure composed of irregularly shaped angular particles of various sizes. The brighter regions are presumably associated with Fe- and Ni-bearing phases, whereas the darker areas correspond to silicate and Mg–silicate components. The fractured morphology and the presence of fine particles may facilitate the access of sulfuric acid to reactive mineral phases during the leaching process.

EDS analysis revealed that the ore was predominantly composed of iron, with significant amounts of magnesium, silicon, and nickel, and minor amounts of aluminum and calcium (Figure S1 and Table S1). The highest standard deviations were observed for Fe (29.93 ± 28.35 wt.%) and Si (16.57 ± 12.41 wt.%), reflecting the presence of mineral phases with markedly different compositions, ranging from quartz (Point 4, Si = 44.33 wt.%) to iron-rich phases (Point 1, Fe = 84.77 wt.%).

Particular attention should be given to the distribution of nickel (Table S2). The standard deviation of the Ni content (3.33 wt.%) exceeds its average concentration (2.79 wt.%), while the measured values range from 1.05 to 10.25 wt.%. Moreover, nickel was detected in 7 of 9 analyzed spots. An exceptionally high Ni concentration was recorded at Spot 2 (10.25 wt.%), which is approximately 4–10 times higher than that observed at the remaining nickel-containing locations (1.05–2.55 wt.%). Nickel was not detected at Spots 1 and 4. Such variations indicate local enrichment of nickel in specific mineral phases and confirm the highly heterogeneous distribution of the metal within the ore.

The elemental composition of the raw ore determined by X-ray fluorescence (XRF) analysis is presented in Table 1.

X-ray fluorescence analysis was used to determine the compositions of the major and trace elements in the ore. Oxygen accounted for 52.56 wt.% of the total mass, confirming the predominance of oxide minerals in the sample. The high oxygen content is associated with the presence of silicate minerals (SiO_2_), iron oxides (Fe_2_O_3_ and/or Fe_3_O_4_), magnesium oxides, and other oxygen-bearing phases. High concentrations of magnesium and silicon were present, reaching 17.16 wt.% and 17.65 wt.%, respectively. These results are consistent with the EDS data and indicate that the ore is predominantly composed of Mg–Si–Fe mineral phases containing nickel.

Iron accounted for 10.11 wt.% of the total composition, indicating the presence of iron-bearing minerals such as magnetite, hematite, and other iron oxides. Sodium, calcium, and aluminum were present at relatively low concentrations (less than 1 wt.%), suggesting the presence of minor amounts of feldspars, plagioclase, or related aluminosilicate minerals.

Nickel (1.0 wt.%), cobalt, and chromium were present at relatively low concentrations; however, their occurrence highlights the metallurgical significance of the ore. Elements such as phosphorus, sulfur, zinc, and copper were detected only in trace amounts (below 0.05 wt.%), indicating the presence of minor impurities or accessory mineral phases within the ore matrix.

Overall, the ore was composed predominantly of oxide and silicate minerals enriched in iron, magnesium, and silicon, which are characteristic of mafic and ultramafic rocks. The presence of nickel, cobalt, and chromium further enhances the economic potential of the deposit as a source of strategic metals for metallurgical applications.

### 3.2. Effects of External Factors on the Adsorption of Nickel

Adsorption studies were conducted using multicomponent model solutions simulating the composition of the pregnant leach solution obtained after ore leaching, which contained 1.5 g/L of Ni^2+^, 0.3 g/L of Co^2+^, and 14 g/L of Mg^2+^. Nickel was selectively recovered using the investigated resins (Figure S2a), whereas the concentrations of cobalt (Figure S2b) and magnesium (Figure S2c) remained essentially unchanged throughout the experiments. These results are consistent with the expected affinity sequence characteristic of this type of chelating resin toward metal ions: Cu^2+^ > Ni^2+^ > Fe^3+^ > Co^2+^ > Zn^2+^ > Mn^2+^ > Mg^2+^ > Ca^2+^ > Al^3+^ [35,50]. Therefore, all subsequent investigations and calculations were focused on Ni(II) ions.

**Figure 2:**
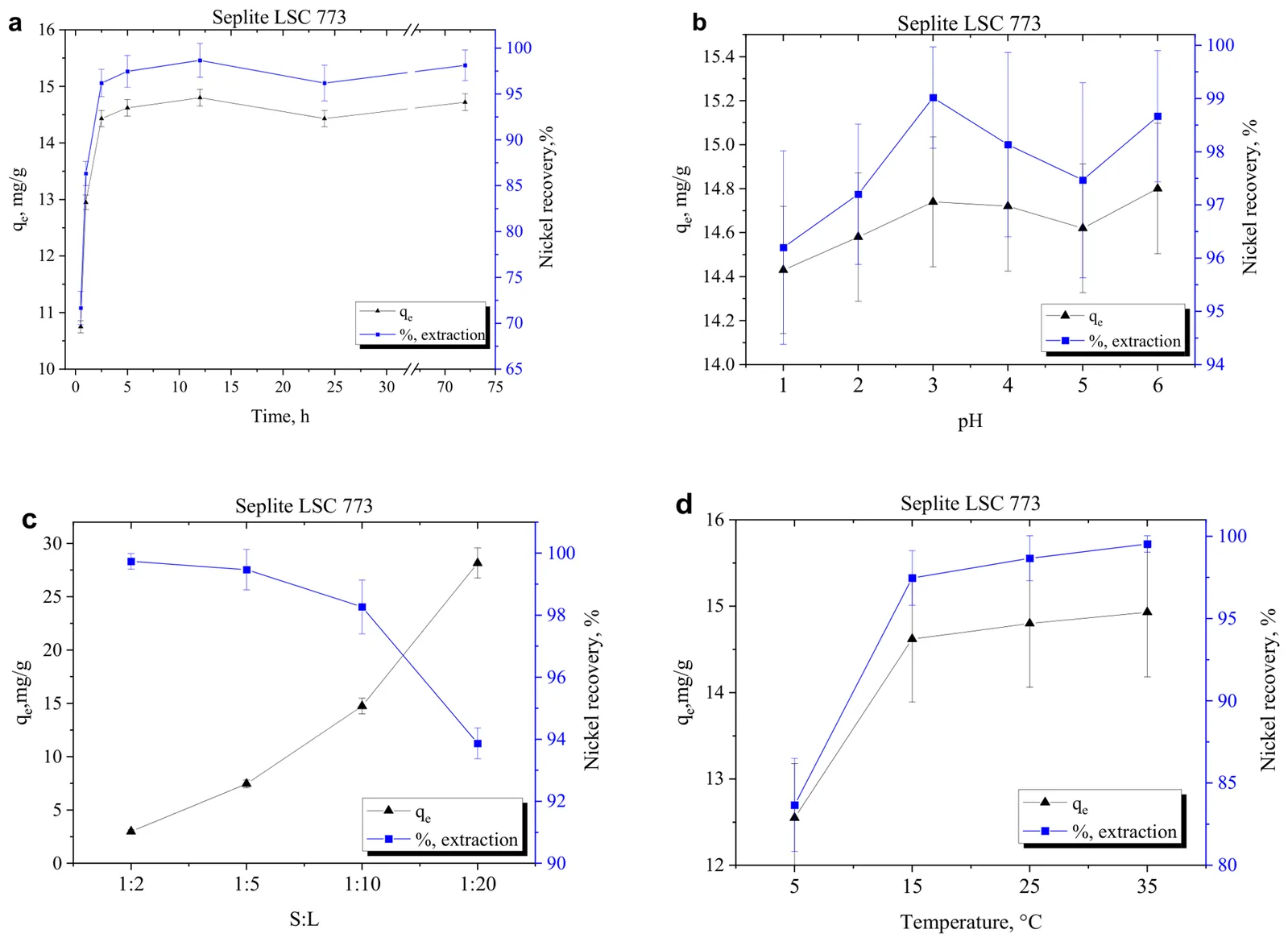
Effects of (**a**) contact time (pH = 5, S:L = 1:10, t = 25 °C); (**b**) pH (S:L = 1:10, t = 25 °C, τ = 5 h); (**c**) S:L ratio (pH = 5, t = 25 °C, τ = 5 h); and (**d**) temperature (pH = 5, S:L = 1:10, τ = 5 h) on q_e_ and the recovery of Ni^2+^ onto Seplite LSC 773.

**Figure 3:**
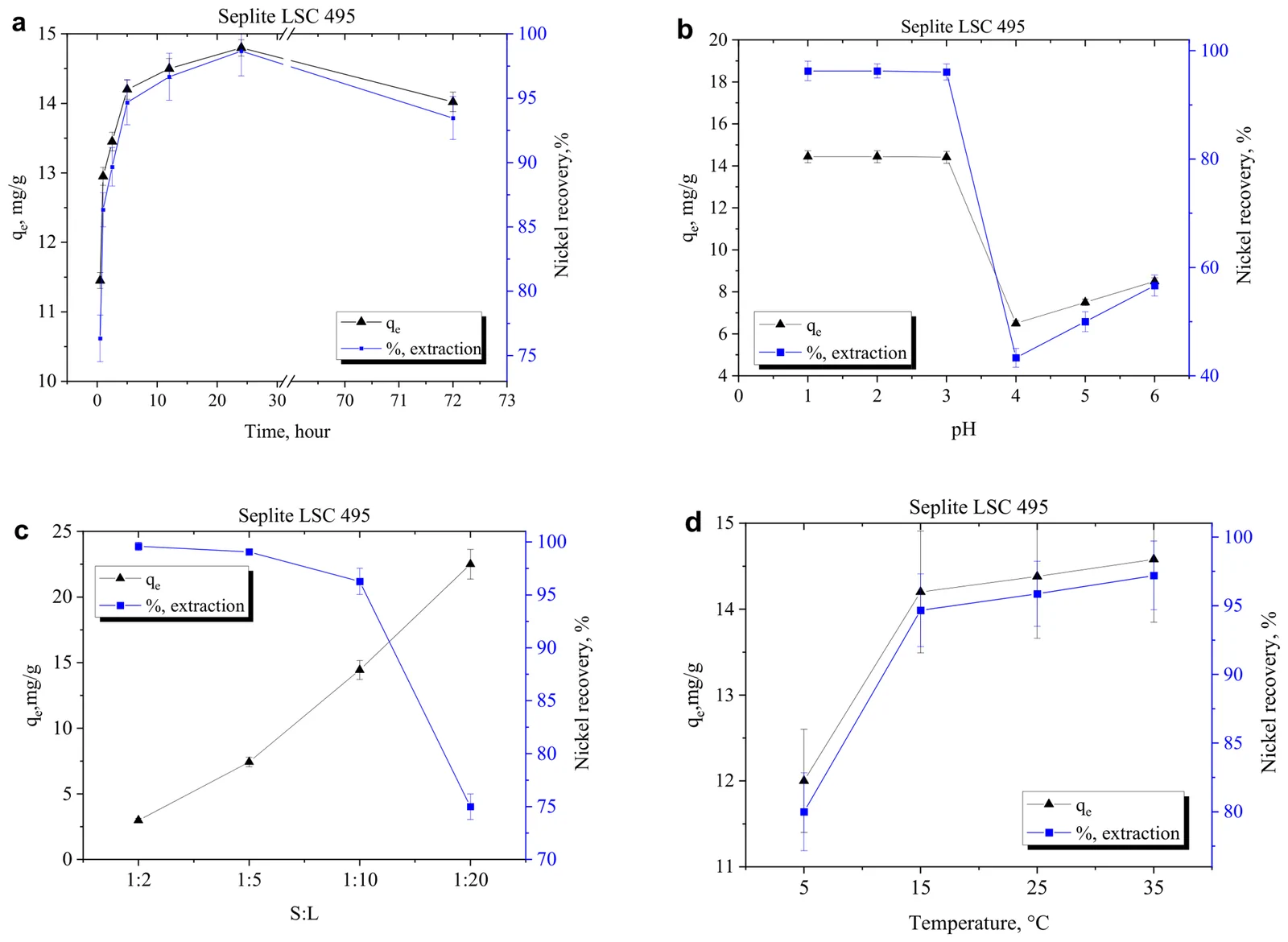
Effects of (**a**) contact time (pH = 5, S:L = 1:10, t = 25 °C); (**b**) pH (S:L = 1:10, t = 25 °C, τ = 5h); (**c**) S:L ratio (pH = 5, t = 25 °C, τ = 5 h); and (**d**) temperature (pH = 5, S:L = 1: 10, τ = 5 h) on q_e_ and the recovery of Ni^2+^ onto Seplite LSC 495.

The effects of contact time, solution pH, solid-to-liquid ratio, and temperature on the sorption of Ni^2+^ ions by Seplite LSC 773 (Figure 2) and Seplite LSC 495 (Figure 3) resins were investigated.

More than 90% of the nickel was recovered within the first 5 h of contact, after which sorption equilibrium was reached (Figure 2a and Figure 3a).

The effect of pH was investigated over the range of 1.0–6.0 to prevent the precipitation of metal hydroxides. A comparative evaluation of Seplite LSC 495 and Seplite LSC 773 ion-exchange resins for nickel recovery from model solutions clearly demonstrated the technological advantage of the LSC 773 resin.

This sorbent exhibited high performance over a broad range of acidities, achieving nickel recovery efficiencies of 96.2–98.7% at pH values of 3.0–6.0. In contrast, LSC 495 showed optimal performance only within a narrow strongly acidic range (pH 1.0–2.0), where the nickel recovery level reached 94.7–96.1%.

The observed differences in the pH dependence of sorption may indicate that the two resins have distinct Ni^2+^ binding mechanisms. For Seplite LSC 495, an acid-dependent ion-exchange mechanism likely predominates, whereas Seplite LSC 773 appears to exhibit a more pronounced chelation-based complexation mechanism, enabling stable nickel recovery over a relatively wide pH range.

The high sorption performance of Seplite LSC 773 at pH 1.5–2.0 is consistent with data in the literature for chelating resins containing N-donor functional groups, which can efficiently recover Ni^2+^ from acidic sulfate solutions [24,36,51].

When the solid-to-liquid ratio (S:L) was varied from 1:2 to 1:20, the optimum value was found to be 1:5, providing nickel recovery efficiencies of 99.1% and 99.5% for Seplite LSC 495 and Seplite LSC 773, respectively. Increasing the sorbent dosage to an S:L ratio of 1:2 resulted in only a marginal improvement in nickel recovery while increasing sorbent consumption levels and associated process costs. Conversely, further increasing the S:L ratio to 1:10 and 1:20 decreased the nickel recovery to approximately 94% and 75% for LSC 773 and LSC 495, respectively.

However, the opposite trend was observed for the adsorption capacity (q_e_, mg/g), which increased with increasing S:L ratio. At lower S:L ratios, the larger amount of resin provides a greater total number of available binding sites relative to the amount of Ni(II) in solution. However, because q_e_ is normalized to the mass of resin, this excess sorbent capacity does not necessarily translate into a higher uptake per gram of resin. As the resin dosage decreases, a greater fraction of the available binding capacity of each resin unit can be utilized, leading to an increase in q_e_. Similar behavior has been reported for Ni^2+^ adsorption on zeolitic materials [52,53].

An increase in temperature from 5 to 35 °C resulted in enhanced sorption performance for both sorbents. The maximum nickel recovery achieved by Seplite LSC 773 reached 97.5% at 25 °C, whereas Seplite LSC 495 exhibited a lower recovery of 94.6% under the same conditions.

These results demonstrate the superior sorption performance of Seplite LSC 773 over Seplite LSC 495 for the recovery of nickel ions from sulfate solutions.

On the basis of the results of the adsorption studies using model solutions, the optimal conditions for nickel recovery were established. For the Seplite LSC 495 resin, the most effective operating parameters were a pH of 1, a contact time of 5 h, a solid-to-liquid ratio (S:L) of 1:5, and a temperature of 25 °C. Under these conditions, nickel recovery reached 94.62 ± 0.21%, with an adsorption capacity (q_e_) of 7.10 ± 0.02 mg/g (Figure S3). For Seplite LSC 773, the optimal performance was achieved at a pH of 3 under the same contact time, S:L ratio, and temperature conditions, resulting in nearly complete nickel recovery (99.36 ± 0.08%) and an adsorption capacity of 7.45 ± 0.01 mg/g.

Nickel recovery from an industrial pregnant leach solution obtained by sulfuric acid leaching was subsequently investigated. The solution composition was as follows: 1.68 g/L of Ni^2+^, 0.33 g/L of Co^2+^, and 32.00 g/L of Mg^2+^. The experiments were conducted at 25 °C, an S:L ratio of 1:5, and a contact time of 300 min (Figure S3). The use of Seplite LSC 495 at a pH of 1 resulted in a nickel recovery of 94.23 ± 0.21%, whereas the use of Seplite LSC 773 at a pH of 3 resulted in a higher recovery of 98.59 ± 0.36%. These values were in close agreement with those obtained for the model solutions, demonstrating the applicability of the investigated sorbents for nickel recovery from real industrial process solutions.

### 3.3. Kinetics

The adsorption kinetics of Ni^2+^ onto Seplite LSC 495 and Seplite LSC 773 were evaluated using pseudo-first-order, pseudo-second-order, chemisorption diffusion, Weber–Morris, Dumwald–Wagner, and Elovich kinetic models (Table 2) to elucidate the adsorption mechanism and identify the rate-limiting steps.

Among all the investigated models, the nonlinear pseudo-second-order equation provided the best description of the experimental data for Seplite LSC 495 (R^2^ = 0.9514, RMSE = 0.2249 mg g^−1^), whereas the nonlinear pseudo-first-order (PFO) model slightly better described the data for Seplite LSC 773 (R^2^ = 0.9699, RMSE = 0.2491 mg g^−1^). For Seplite LSC 773, the corresponding pseudo-second-order (PSO) fit was also satisfactory (R^2^ = 0.9492, RMSE = 0.3238 mg g^−1^). Thus, the kinetics were not adequately described by a single model common to both sorbents. Moreover, agreement with the pseudo-second-order model was not considered independent evidence of a chemisorption-controlled mechanism. In contrast, for Seplite LSC 495, the pseudo-first-order model showed a lower goodness of fit (R^2^ = 0.8584, RMSE = 0.3836 mg g^−1^) than the pseudo-second-order model, indicating that the adsorption kinetics of this resin are better represented by the latter model [40,41].

The fitted kinetic parameters also differed between the two sorbents. The nonlinear pseudo-second-order rate constants were k_2_ = (9.42 ± 1.31) × 10^−3^ g mg^−1^ min^−1^ for Seplite LSC 495 and (6.24 ± 0.97) × 10^−3^ g mg^−1^ min^−1^ for Seplite LSC 773. The corresponding calculated equilibrium capacities were 14.36 ± 0.16 and 15.00 ± 0.23 mg g^−1^, respectively. For the nonlinear pseudo-first-order model, k_1_ values of 0.0547 ± 0.0061 and 0.0428 ± 0.0025 min^−1^ were obtained for Seplite LSC 495 and LSC 773, respectively. Because the PFO and PSO models showed different relative goodness of fit for the two resins, the rate constants should be interpreted within the respective models rather than used alone to infer differences in the intrinsic adsorption mechanism or active-site accessibility.

The chemisorption diffusion model showed reasonably good agreement with the experimental data (R^2^ = 0.8800 for Seplite LSC 495 and R^2^ = 0.8295 for Seplite LSC 773), but provided a poorer fit than the best-performing PFO or PSO model for each resin. These results suggest that diffusion–adsorption effects may contribute to the overall uptake kinetics but do not independently establish chemisorption as the dominant rate-limiting mechanism. The higher chemisorption diffusion constant obtained for Seplite LSC 495 (K_CD_ = 9.81 mg g^−1^ min^−0^·^5^) than that for Seplite LSC 773 (6.91 mg g^−1^ min^−0^·^5^) indicates a difference in the empirical diffusion–chemisorption response of the two resins. However, this difference should not be interpreted as direct evidence of a greater contribution of a specific surface-reaction step.

The Dumwald–Wagner model showed moderate agreement with the experimental data (R^2^ = 0.8009–0.8894). Although these results suggest that diffusion-related mass transfer may contribute to the overall adsorption kinetics, the model does not provide sufficient evidence to identify diffusion as the sole rate-limiting step.

The Weber–Morris intraparticle diffusion model exhibited poor linearity (R^2^ = 0.2330–0.2826), indicating that intraparticle diffusion alone cannot describe the adsorption kinetics. Therefore, the kinetic parameters derived from this model were interpreted cautiously and were not used to assign intraparticle diffusion as the rate-controlling mechanism. Similarly, the Elovich equation yielded relatively poor fits (R^2^ = 0.5402–0.6113), indicating that this model described the experimental kinetic profiles less satisfactorily than the PFO and PSO models. Consequently, the Elovich parameters were not discussed further.

Overall, the results of the kinetic analysis indicate that Ni^2+^ uptake by the two chelating resins is not adequately described by a single common kinetic model. The PSO model provided the best fit for Seplite LSC 495, whereas the PFO model slightly outperformed the PSO model for Seplite LSC 773. The comparatively poorer performance of the Weber–Morris, Dumwald–Wagner, Elovich, and chemisorption-diffusion models indicates that no single diffusion- or surface-reaction-based description fully accounts for the observed kinetic behavior. Accordingly, the kinetic modelling is consistent with a multistep adsorption process involving both Ni^2+^ binding at chelating sites and mass-transfer processes, without assigning a unique rate-controlling mechanism solely on the basis of model fitting.

### 3.4. Isotherms

**Figure 4:**
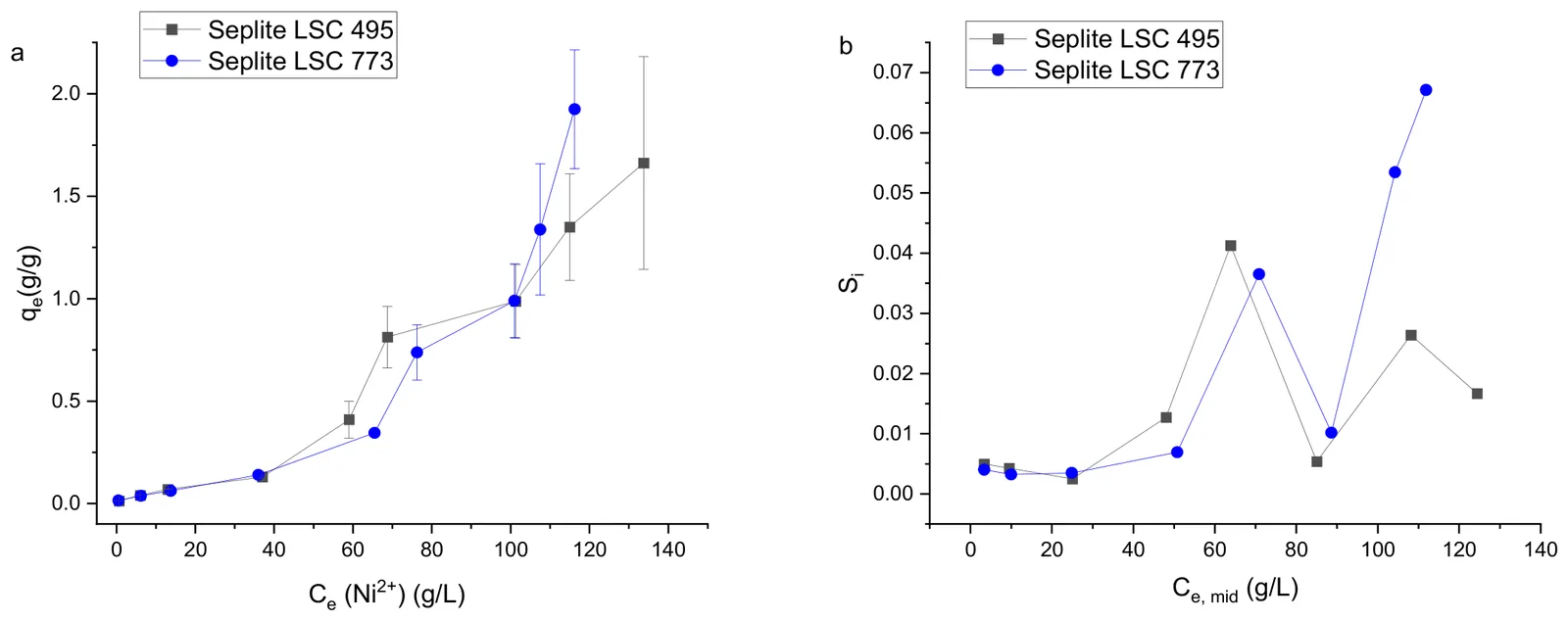
(**a**) Experimental adsorption isotherms and (**b**) local adsorption slope of Ni^2+^ extraction from model solutions onto Seplite LSC 495 and Seplite LSC 773 (pH = 3, S:L = 1:10, t = 25 °C, τ = 5 h).

To construct equilibrium adsorption isotherms for Ni^2+^ onto Seplite LSC 495 and Seplite LSC 773 (Figure 4a), adsorption was performed in an initial Ni^2+^ concentration range of 2 to 300 g/L. Further increases in concentration were considered to be above the solubility limit of the salt. At low equilibrium concentrations, only limited Ni^2+^ uptake was observed, indicating relatively weak interactions between hydrated nickel ions and the initially available chelating functional groups. However, above approximately 60–80 g L^−1^, the equilibrium uptake increased sharply, producing a characteristic S-shaped isotherm.

At the highest investigated Ni^2+^ concentrations, the solution-depletion-based calculation yielded apparent q_e_ values approaching 2 g g^−1^. Given the unusually high magnitude of these values for commercial chelating resins, they should be interpreted with caution. In the present study, q_e_ was determined indirectly from the difference between the initial and equilibrium liquid-phase Ni^2+^ concentrations rather than by direct quantification of Ni in the loaded resin. Furthermore, no distinct saturation plateau was reached within the investigated concentration range. Therefore, the highest experimental q_e_ values represent apparent equilibrium uptake under the specific batch conditions employed and should not be interpreted as the intrinsic maximum adsorption capacity (q_max_) of the resins.

Both sorbents exhibited similar adsorption behaviors characterized by a pronounced sigmoidal (S-type) profile according to the Giles classification (Figure 4a) [54].

According to the Giles classification, S-type isotherms are characterized by a relatively low initial slope followed by a progressively steeper uptake region [55]. Although such profiles are frequently associated with cooperative adsorption, the sigmoidal shape alone does not provide direct evidence of cooperative interactions between adjacent adsorption sites [56]. For chelating ion-exchange resins, the observed concentration-dependent adsorption behavior may reflect several effects, including changes in the accessibility of chelating sites, conformational rearrangement or swelling of the polymer matrix during metal loading, and changes in the local environment of the functional groups.

To provide a quantitative assessment of the experimentally observed S-type behavior, the local adsorption slope ($S_{i}$) was calculated as follows:

(14) $$S_{i}=\frac{q_{e,i+1}-q_{e,i}}{C_{e,i+1}-C_{e,i}}$$

where $q_{e}$ and $C_{e}$ are the equilibrium adsorption capacity and equilibrium Ni^2+^ concentration, respectively. For both sorbents, the initial slope values (Figure 4b) remained low (approximately 0.003–0.005 g g^−1^ L g^−1^), confirming the limited affinity of the resins for Ni^2+^ at low equilibrium concentrations. As the concentration increased, the local slope increased markedly, peaking in the concentration range of approximately 60–70 g L^−1^ for Seplite LSC 495 and above 100 g L^−1^ for Seplite LSC 773.

For both sorbents, the local slope was relatively low in the initial concentration region and increased as the isotherms entered their steeper regions (Figure 4b). This trend quantitatively describes the concentration-dependent change in the steepness of the experimental isotherms and is consistent with their S-type shape according to the Giles classification. However, because an increasing local slope is inherently expected for the ascending region of a sigmoidal isotherm, this analysis is not considered independent evidence of cooperative interactions between adsorption sites.

Therefore, the selection of adsorption isotherm models was based on experimentally observed adsorption behavior rather than on conventional empirical practice. Because the experimental isotherms did not satisfy the fundamental assumptions of homogeneous monolayer adsorption, models capable of describing heterogeneous adsorption systems were selected. Specifically, the Freundlich [48] model was employed to evaluate the energetic heterogeneity of the adsorption sites, the Toth [49] model was used to describe adsorption on heterogeneous surfaces with nonuniform adsorption energies, the Sips [50] model was used to account for heterogeneous adsorption approaching saturation at elevated equilibrium concentrations, and the Redlich–Peterson [51] model was used as a versatile hybrid equation for describing nonideal adsorption systems exhibiting deviations from both Langmuir and Freundlich behaviors.

The sorbents were not saturated within the investigated concentration range because the solubility limit of the nickel salt restricted further increases in the initial Ni^2+^ concentration. Therefore, the maximum adsorption capacity cannot be experimentally determined under these conditions. Consequently, adsorption models requiring reliable estimation of the saturation capacity ($q_{m}$), such as the Sips, Toth, and Redlich–Peterson equations, cannot provide physically meaningful values of $q_{m}$. Nevertheless, these models were fitted to the experimental data for completeness, and the corresponding fitting parameters and statistical goodness-of-fit criteria are presented in the Supplementary Information (Table S4).

The nonlinear fitting results demonstrate that the Freundlich model provides the most physically meaningful description of the experimental equilibrium data for both sorbents. The high goodness-of-fit indicates that Ni^2+^ adsorption occurs predominantly on energetically heterogeneous binding sites rather than on a homogeneous surface. This observation is fully consistent with the structural characteristics of chelating ion-exchange resins, in which functional groups are distributed throughout a cross-linked polymer matrix and exhibit different local coordination environments.

Taken together, the Freundlich fitting and the S-type shape of the experimental isotherms indicate nonideal, concentration-dependent Ni^2+^ uptake by the chelating resins and are consistent with heterogeneity of the adsorption environment. The sigmoidal profile may additionally reflect changes in site accessibility or in the local environment of the functional groups as metal loading increases. However, the present equilibrium data do not allow these effects to be distinguished from true cooperative interactions between adjacent adsorption sites. Therefore, cooperative adsorption is considered a possible interpretation of the S-type behavior rather than an experimentally demonstrated mechanism.

### 3.5. FTIR Spectra of the Spent Adsorbents and Mechanistic Interpretation of Ni^2+^ Adsorption

**Figure 5:**
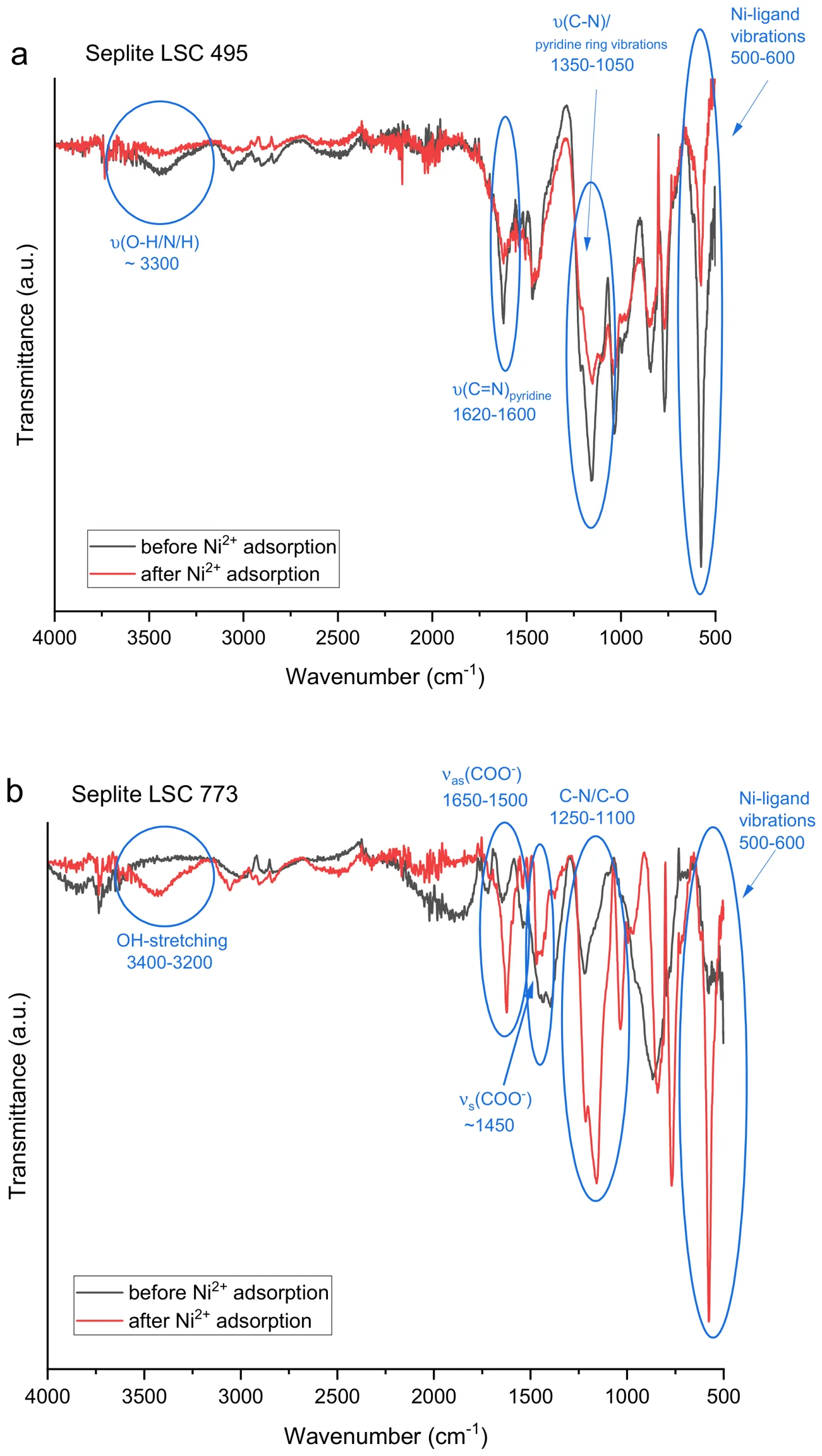
FTIR spectra of (**a**) Seplite LSC 495 and (**b**) Seplite LSC 773 before and after Ni(II) sorption.

The FTIR spectra of Seplite LSC 773 and Seplite LSC 495 before and after Ni(II) adsorption are presented in Figure 5. In both cases, the overall spectral profiles remained essentially unchanged after adsorption, indicating that the polymeric matrices of the resins were preserved during sorption. However, noticeable changes in the position, shape, and relative intensity of several characteristic absorption bands were observed (Table S5), suggesting modification of the chemical environment of the chelating functional groups upon interaction with Ni(II).

With respect to Seplite LSC 773, which contains iminodiacetate functional groups, the most pronounced spectral changes occurred in the 1650–1300 cm^−1^ region. The band that is predominantly related to the asymmetric stretching vibration of the carboxylate group [57] shifted from 1648.4 to 1622.8 cm^−1^, whereas the band associated with the symmetric carboxylate vibration [58] shifted from 1459.1 to 1469.7 cm^−1^. Consequently, the separation between the asymmetric and symmetric COO^−^ stretching bands decreased from approximately 189 to 153 cm^−1^, indicating a substantial change in the coordination environment of the carboxylate groups after Ni(II) adsorption [58,59]. Additional shifts observed at 1137.8 → 1152.3 cm^−1^ and 1038.0 → 1033.9 cm^−1^ correspond to vibrations involving C–N and C–O bonds of the iminodiacetate ligand [60], suggesting that both nitrogen and oxygen donor atoms participate in metal binding. Changes in the lower-frequency region (868–769 cm^−1^) further indicate structural perturbation of the functionalized polymer matrix following complex formation [57,61].

In contrast, Seplite LSC 495, which is functionalized with bispicolylamine groups, exhibited considerably smaller shifts in most of the characteristic bands. The vibrations assigned to the pyridine ring and tertiary amine groups remained nearly unchanged, with peak displacements generally less than 2 cm^−1^. The most noticeable changes were observed for the bands located at 1106.9 → 1097.8 cm^−1^ and 842.5 → 849.2 cm^−1^, which are attributed to C–N stretching and pyridine ring vibrations [62,63], respectively. Although these positional shifts are more modest than those observed for Seplite LSC 773, they were accompanied by distinct changes in band shape and relative intensity, indicating perturbation of the bispicolylamine donor environment after Ni(II) adsorption [64,65].

The different spectral responses of the two sorbents reflect their distinct coordination chemistries. In Seplite LSC 773, the significant shifts in the carboxylate vibrations indicate that oxygen-containing donor groups play a dominant role in Ni(II) coordination, along with the amine nitrogen of the iminodiacetate ligand. Conversely, the relatively small positional changes observed for Seplite LSC 495 suggest that Ni(II) binding is associated mainly with the nitrogen donor atoms of the bispicolylamine ligand, resulting primarily in the redistribution of electron density rather than substantial changes in the vibrational frequencies of the pyridine rings.

Overall, the FTIR results were consistent with the formation of coordination complexes between Ni(II) ions and the chelating functional groups of both resins, confirming that the polymeric matrices remained structurally stable throughout adsorption.

Based on the combined kinetic, equilibrium, and spectroscopic results, Ni^2+^ adsorption by Seplite LSC 495 and Seplite LSC 773 can be interpreted as a multistep process involving transport of hydrated Ni^2+^ ions into the polymer matrix followed by their binding to the chelating functional groups. Initially, Ni^2+^ ions are transferred from the bulk solution to the resin surface and diffuse through the hydrated polymer network toward accessible binding sites. Interaction with the functional groups may involve partial dehydration of Ni^2+^ followed by coordination with donor atoms of the chelating ligands [62].

The nature of the binding sites differs between the two resins. For Seplite LSC 773, the iminodiacetate groups provide carboxylate oxygen and amine nitrogen donor atoms for Ni^2+^ coordination [34], whereas the bispicolylamine groups of Seplite LSC 495 provide pyridine and amine nitrogen donor sites [64]. The FTIR spectral changes observed after Ni^2+^ adsorption are consistent with the involvement of these respective functional groups. The equilibrium behavior further indicates that these binding sites are not energetically equivalent: the concentration-dependent uptake and the superior Freundlich fit are consistent with a heterogeneous distribution of Ni^2+^ binding environments within the polymer matrix. Thus, Ni^2+^ retention is most reasonably attributed to specific metal–ligand interactions occurring at chemically and energetically nonequivalent chelating sites rather than to nonspecific physical adsorption alone.

The kinetic results complement this interpretation by indicating that the relative contributions of the individual adsorption steps differ between the two resins. LSC 495 was better described by the nonlinear PSO model, whereas LSC 773 showed slightly better agreement with the nonlinear PFO model. At the same time, the poor Weber–Morris fits indicate that intraparticle diffusion alone cannot account for the observed uptake kinetics. Taken together, the results support a mechanistic picture in which Ni^2+^ transport through the hydrated resin matrix is followed by coordination at chemically specific and energetically heterogeneous chelating sites, with the relative kinetic contribution of these steps depending on the functional-group chemistry and polymer environment of each resin.

### 3.6. Desorption and Regeneration Studies

#### 3.6.1. Eluent Concentration Optimization

The practical applicability of chelating ion-exchange resins is determined not only by their adsorption capacity but also by their regeneration efficiency and stability during repeated adsorption–desorption cycles [66,67].

As an initial step, the effect of the concentration of the desorbing agent (H_2_SO_4_) on the desorption efficiency of Ni^2+^ was investigated over a concentration range of 10–30 wt.% (Figure S4). The obtained results revealed a pronounced nonlinear dependence of the desorption efficiency on the sulfuric acid concentration. At 10 wt.% H_2_SO_4_, only limited desorption is achieved, with Ni^2+^ recoveries of 35.31% for Seplite LSC 495 and 30.88% for Seplite LSC 773. Increasing the acid concentration to 20 wt.% significantly enhanced the desorption process, resulting in maximum recoveries of 42.37% and 41.62% for Seplite LSC 495 and Seplite LSC 773, respectively.

Interestingly, a further increase in the sulfuric acid concentration to 30 wt.% decreased the desorption efficiency to 36.72% for Seplite LSC 495 and to 33.57% for Seplite LSC 773. Several factors may contribute to this behavior, including the increased ionic strength and reduced water activity of concentrated sulfuric acid solutions, which can suppress ion exchange and mass transfer. In addition, highly acidic conditions may induce partial contraction of the polymer matrix, thereby reducing the accessibility of internal chelating sites and hindering the diffusion of coordinated Ni^2+^ ions from the resin interior to the bulk solution [68]. Similar nonmonotonic effects of acid concentration on the regeneration efficiency of chelating ion-exchange resins have been reported previously and are commonly attributed to changes in both polymer swelling behavior and ion transport kinetics [39].

Among the investigated H_2_SO_4_ concentrations, 20 wt.% provided the highest Ni(II) desorption efficiency and was therefore selected for the subsequent regeneration experiments. However, the maximum desorption efficiencies of 42.37% for Seplite LSC 495 and 41.62% for Seplite LSC 773 indicate that regeneration remained incomplete under the investigated conditions. Thus, 20 wt.% H_2_SO_4_ should be regarded as the most effective condition among those tested rather than as an industrially optimized regeneration condition.

The relatively low desorption efficiencies obtained under the most effective investigated conditions indicate that a substantial fraction of the adsorbed Ni^2+^ remained associated with the resin after acid treatment. This incomplete regeneration may reflect the strong interaction of Ni^2+^ with the chelating functional groups and/or limitations in ion transport and accessibility of internal binding sites. Further studies are required to distinguish the contributions of these factors and to optimize the regeneration procedure.

#### 3.6.2. Multistage Desorption and Reusability

**Figure 6:**
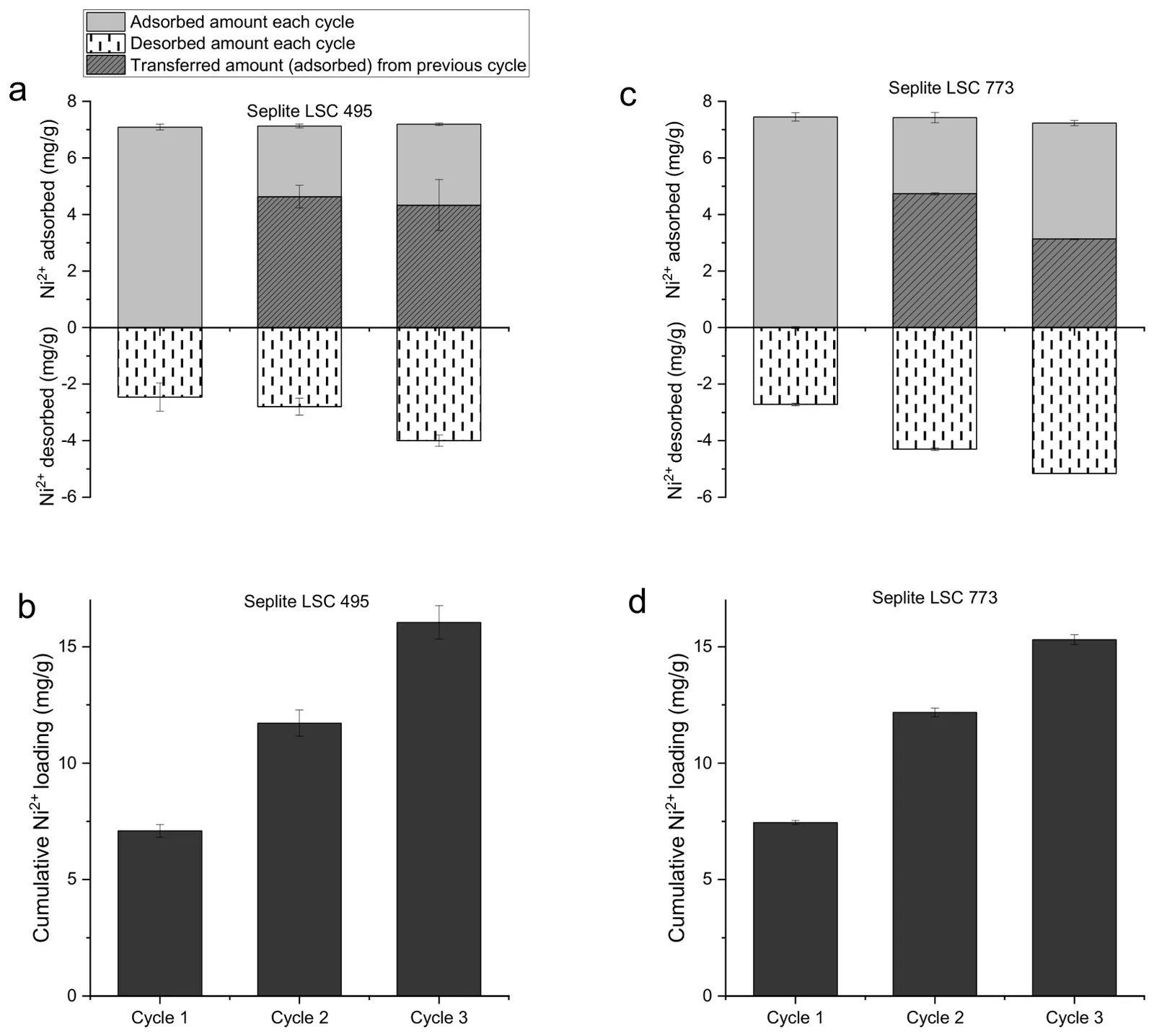
Consecutive Ni^2+^ adsorption–desorption cycles and Ni^2+^ net uptakes for Seplite LSC 495 (**a**,**b**) and Seplite LSC 773 (**c**,**d**).

The regeneration performance levels of Seplite LSC 495 and Seplite LSC 773 under the optimized desorption conditions (20 wt.% H_2_SO_4_) were evaluated through three consecutive adsorption–desorption cycles (Figure 6). In addition to the adsorption and desorption capacities measured during each cycle, the cumulative amount of residual Ni^2+^ remaining on the sorbent after regeneration was calculated to assess the regeneration behaviors of the chelating resins during repeated use.

As shown in Figure 6a,c, both sorbents exhibited only minor changes in their adsorption capacity over the three consecutive cycles. For Seplite LSC 495, the adsorption capacity varied from 7.09 to 7.19 mg g^−1^, whereas that of Seplite LSC 773 slightly decreased from 7.45 to 7.23 mg g^−1^ after the third cycle. Within the investigated number of cycles, these results indicate that the adsorption performance remained relatively stable and that no pronounced deterioration of the sorbent was observed.

The desorption behavior tended to differ. For both sorbents, the amount of Ni^2+^ recovered during regeneration gradually increased with increasing cycle number. The desorbed amount increased from 2.46 to 4.00 mg g^−1^ for Seplite LSC 495 and from 2.72 to 5.16 mg g^−1^ for Seplite LSC 773 between the first and third cycles. This behavior suggests that regeneration became progressively more effective during repeated adsorption–desorption treatments. One possible explanation is a gradual increase in the accessibility of internal chelating sites or diffusion pathways within the polymer matrix during repeated regeneration; however, additional structural characterization is needed to verify this hypothesis.

The cumulative Ni^2+^ loading after each adsorption step is presented in Figure 6b,d. Because desorption remained incomplete under the selected regeneration conditions, a fraction of the adsorbed Ni^2+^ was retained within the sorbent after each cycle, resulting in a progressive increase in cumulative Ni^2+^ loading. Consequently, the cumulative loading increased from 7.09 to 16.15 mg g^−1^ for Seplite LSC 495 and from 7.45 to 15.09 mg g^−1^ for Seplite LSC 773 over the three adsorption–desorption cycles.

Within the three cycles investigated, both sorbents retained a substantial fraction of their adsorption capacity, with only minor changes in Ni(II) uptake. However, these results should be regarded as a preliminary assessment of reusability rather than evidence of long-term operational stability, particularly because the regeneration step remained incomplete. Longer-term cycling experiments and improved regeneration protocols will be required to establish the practical durability of the resins.

### 3.7. Proposed Flowsheet for the Integrated Processing of Oxidized Nickel Ores

**Figure 7:**
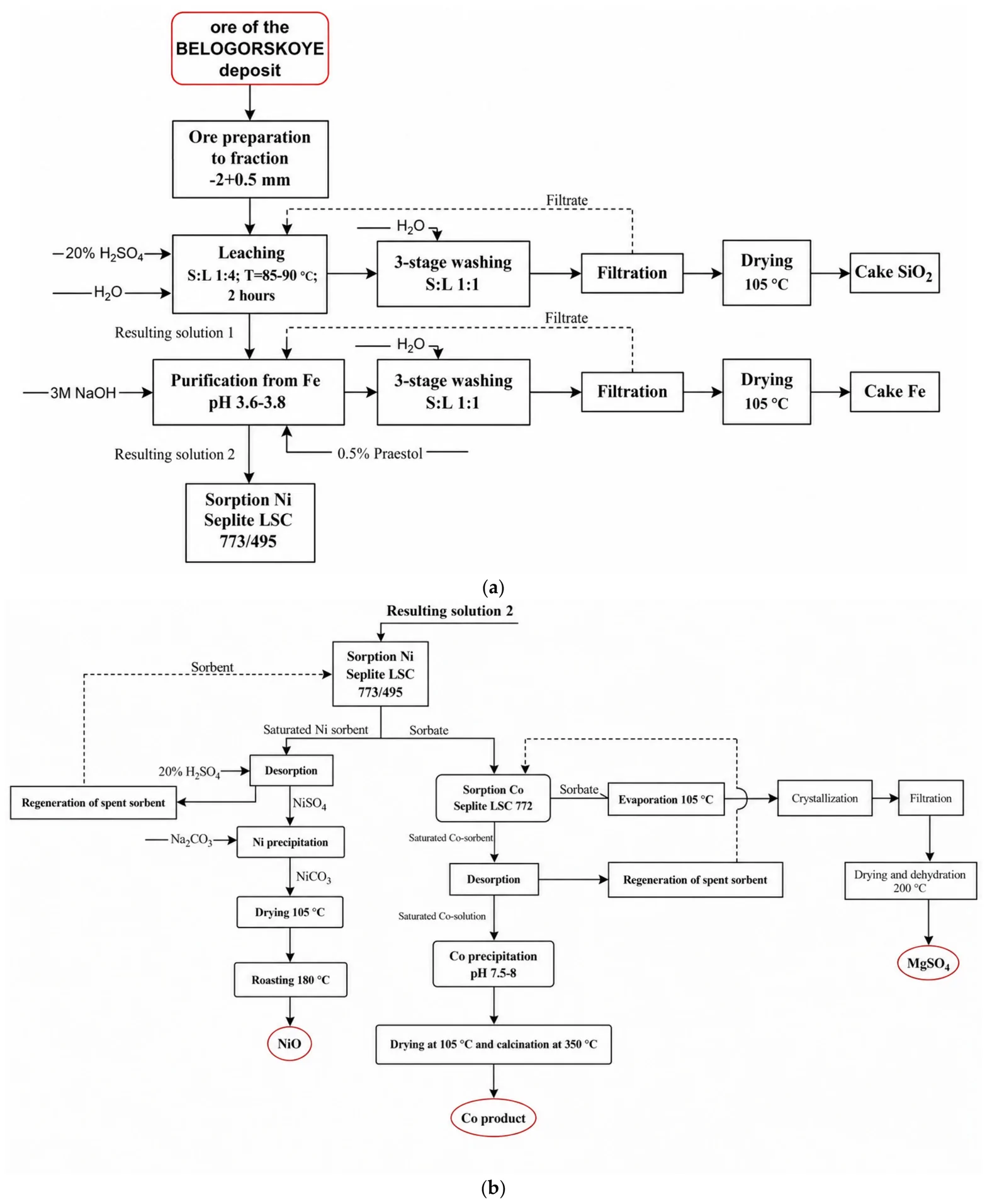
Proposed process flowsheet for the integrated recovery of valuable components from oxidized nickel ore: (**a**) hydrometallurgical processing; (**b**) sorption–desorption separation and product recovery.

On the basis of the experimental results obtained in this study, a conceptual flowsheet for the integrated processing of oxidized nickel ores is proposed (Figure 7). The scheme combines the leaching of sulfuric acid, the selective separation of metals by ion-exchange adsorption, and the production of recovered products.

The process begins with sulfuric acid leaching of the finely ground ore to produce a pregnant leach solution containing Ni^2+^, Co^2+^, Mg^2+^, and Fe^3+^ ions. Following solid–liquid separation and multistage washing of the leach residue, a silica-rich solid product is obtained.

The pregnant leach solution is subsequently subjected to controlled neutralization to remove iron as a precipitate. The purified solution is then directed toward the selective recovery of valuable base metals.

The key stage of the proposed flowsheet is the selective adsorption of nickel using the chelating ion-exchange resins Seplite LSC 495 and Seplite LSC 773. Owing to their high selectivity toward Ni^2+^, these sorbents enable preferential nickel recovery from the multicomponent sulfate solution, while most of the cobalt and magnesium remain in the raffinate. This selective separation process minimizes the transfer of accompanying components to downstream processing stages and facilitates the production of a high-purity nickel product.

After saturation with nickel, the resin is regenerated using sulfuric acid, producing a concentrated nickel sulfate solution. The resulting eluate is subsequently processed to obtain nickel oxide as the final nickel product.

The remaining raffinate after nickel recovery, which is enriched in cobalt and magnesium, is subjected to further separation. Cobalt can be recovered using the chelating resin Seplite LSC 772, followed by the desorption and precipitation of a cobalt-containing product. Magnesium is recovered from the remaining solution through evaporation and crystallization to produce commercial magnesium sulfate.

To provide a quantitative representation of the proposed process, a material balance was established for the principal stages of the flowsheet based on the actual material streams and products obtained during ore processing (Table S6). The proposed process includes sulfuric acid leaching, separation of the silica-rich cake, iron removal, selective Ni recovery followed by desorption and NiO production, and subsequent separation of Co and Mg, with Mg recovered as MgSO_4_·7H_2_O.

The material balance was calculated on the basis of 1000 g of initial ore containing 10 g of Ni and 172 g of Mg. A total of 8.64 g of Ni was transferred to the pregnant leach solution, corresponding to a recovery of 86.4%, which is close to the experimentally determined value of 84.0 ± 1.8% reported in this study. According to the material balance, subsequent processing yielded approximately 11.0 g of dry NiO and 1612 g of MgSO_4_·7H_2_O.

The Co-containing product included in the material balance was experimentally obtained by treating the sorbate with MgO and H_2_O_2_. However, the proposed conceptual flowsheet considers selective Co recovery using Seplite LSC 772, followed by desorption and precipitation of a Co-containing product, as a prospective alternative for further development of the process. The mother liquor generated after Ni precipitation and containing Na_2_SO_4_ is proposed to be directed to a separate purification stage for subsequent removal or recovery of sodium sulfate.

Thus, the material balance reflects the experimentally obtained processing results, whereas the technological flowsheet represents a conceptual framework for further development of the process. The proposed use of Seplite LSC 772 should therefore be regarded as a prospective approach for optimization of the cobalt recovery stage and requires additional experimental validation.

Thus, in the proposed process, selective ion-exchange adsorption functions not only as a purification step but also as the central unit for the extraction, separation, and concentration of valuable metals. Integration of this approach enables the sequential production of nickel oxide, a cobalt-containing product, and magnesium sulfate from a single pregnant leach solution. Furthermore, regeneration of the ion-exchange resins allows their repeated use within the process, contributing to improved resource efficiency and process sustainability.

Notably, the proposed flowsheet is conceptual in nature and was developed based on the experimental findings of this study. Further pilot-scale investigations are needed to validate the industrial applicability of this framework, including evaluation of the long-term stability of the ion-exchange resins, the regeneration efficiency over multiple operating cycles, and the overall technoeconomic performance of the integrated process.

### 3.8. Comparative Assessment of Ni(II) Recovery Systems

To place the performance of Seplite LSC 495 and Seplite LSC 773 in the context of previously reported Ni(II) recovery systems, the results of the present study were compared with those obtained using representative chelating ion-exchange resins and alternative sorbents reported in the literature (Table 3). The selected systems include commercial iminodiacetate- and bispicolylamine-functionalized resins, as well as biochar-, zeolite-, and MnO_2_-based sorbents. Because these studies differ substantially in feed composition, Ni concentration, pH, solid-to-liquid ratio, and operating mode, direct comparison based solely on adsorption capacity or percentage recovery would not be appropriate. Therefore, the comparison considers Ni(II) recovery, selectivity in multicomponent media, operating conditions, regeneration behavior, and applicability to complex hydrometallurgical solutions.

Among the previously reported systems, commercial chelating resins are the most relevant for comparison with the resins investigated in this study. Dowex M4195, containing bispicolylamine functional groups similar to those of LSC 495, achieved more than 99% recovery of Ni and Co from laterite-derived solutions and pulps after five counter-current stages, with a maximum loading of 100 g Ni kg^−1^ resin. Similarly, iminodiacetate-functionalized Amberlite IRC 748 and Purolite S930 have demonstrated effective Ni recovery from multicomponent sulfate media, although co-recovery of other metals and competition for active sites may affect Ni selectivity. Lewatit MonoPlus TP 220, another bispicolylamine-functionalized resin, has also been successfully applied to acidic solutions and regenerated using H_2_SO_4_, although an equilibrium time of 72 h was reported. In comparison, the Seplite resins investigated in the present study achieved high Ni(II) recovery within a substantially shorter contact time of 5 h: LSC 773 reached 99.36 ± 0.08% recovery from the model solution and 98.59 ± 0.36% from the industrial leach solution, whereas LSC 495 achieved 94.62 ± 0.21% and 94.23 ± 0.21%, respectively.

A particularly relevant feature of the present study is that both resins were evaluated not only in model solutions but also in an actual sulfate leach solution containing high concentrations of accompanying ions. Under these conditions, LSC 773 maintained high Ni(II) recovery in the presence of Co(II) and a substantial excess of Mg(II), whereas LSC 495 remained effective under strongly acidic conditions. Alternative low-cost sorbents, including biochars, modified rice husk, Na-mordenite, and MnO_2_, have shown appreciable Ni(II) uptake; however, most were evaluated using model or comparatively dilute solutions and, in some cases, required near-neutral or alkaline pH and elevated temperatures. Consequently, their reported adsorption capacities or recovery efficiencies cannot be directly compared with those obtained for the highly saline and chemically complex sulfate leachate investigated in the present study.

Overall, the comparison indicates that the principal advantage of the present system lies not in exceptionally high intrinsic adsorption capacity but rather in the combination of high Ni(II) recovery, selectivity in a Mg-rich multicomponent sulfate matrix, operation under acidic conditions, and demonstrated applicability to an actual ore-leaching solution. Of the two investigated resins, LSC 773 showed the most favorable overall performance, whereas LSC 495 represents a potentially useful alternative for more strongly acidic feeds. Nevertheless, the incomplete Ni(II) desorption observed with H_2_SO_4_, reaching only approximately 42% under the investigated conditions, remains an important limitation, particularly in comparison with systems for which more efficient regeneration has been reported. Further optimization of the regeneration step, together with validation under continuous-flow or column conditions, is therefore required before the performance demonstrated at the batch scale can be translated into a practical hydrometallurgical process.

## 4. Conclusions

This work provides a comprehensive evaluation of the commercial chelating ion-exchange resins Seplite LSC 495 and Seplite LSC 773 for the selective recovery of Ni(II) from sulfate solutions representative of oxidized nickel ore leachates. Under optimized conditions, Seplite LSC 773 achieved 99.36 ± 0.08% Ni(II) recovery from the model solution and 98.59 ± 0.36% from the industrial leach solution, whereas the corresponding values for Seplite LSC 495 were 94.62 ± 0.21% and 94.23 ± 0.21%, respectively. The high Ni(II) recovery maintained in the presence of Co(II) and a substantial excess of Mg(II) demonstrates the suitability of these chelating resins, particularly LSC 773, for selective Ni recovery from complex sulfate process streams.

Kinetic analysis showed that the adsorption behavior of the two resins could not be represented by a single common kinetic model. Nonlinear fitting indicated that the pseudo-second-order model provided a slightly better fit for LSC 495 (R^2^ = 0.9514), whereas the pseudo-first-order model slightly better described LSC 773 (R^2^ = 0.9699). The equilibrium data exhibited a Giles S-type profile without reaching a distinct saturation plateau within the investigated concentration range. The Freundlich model provided the most appropriate description of the equilibrium data, consistent with adsorption on energetically heterogeneous binding sites. The S-type classification and local slope analysis were therefore used as descriptive characteristics of the experimental isotherm shape rather than as independent evidence of cooperative adsorption. Furthermore, the unusually high apparent values obtained at the highest Ni(II) concentrations should not be interpreted as intrinsic maximum adsorption capacities without independent verification of Ni loading in the solid phase.

FTIR analysis revealed changes in the characteristic bands associated with the chelating functional groups after Ni(II) adsorption, supporting their involvement in Ni(II) binding. Taken together with the kinetic and equilibrium results, these observations are consistent with specific interactions between Ni(II) and the functional groups of the resins; however, the present data do not justify the assignment of a unique adsorption mechanism or rate-controlling step solely on the basis of kinetic model fitting.

Regeneration remains one of the principal limitations identified in this study. Among the conditions investigated, 20 wt.% H_2_SO_4_ provided the highest Ni(II) desorption efficiency, although only approximately 42% of the adsorbed Ni was desorbed. Adsorption capacity changed only slightly over three consecutive adsorption–desorption cycles; however, the incomplete desorption and limited number of cycles preclude conclusions regarding long-term resin stability. Further work should therefore focus on optimizing the eluent composition and operating conditions, evaluating multistage regeneration strategies, and conducting extended cyclic testing.

Beyond batch adsorption performance, the experimental results were incorporated into a conceptual hydrometallurgical flowsheet integrating sulfuric acid leaching, selective Ni recovery, subsequent Co separation, and MgSO_4_·7H_2_O production. On the basis of 1000 g of initial ore containing 10 g of Ni and 172 g of Mg, 8.64 g of Ni was transferred to the pregnant leach solution, corresponding to 86.4% recovery, and subsequent processing yielded approximately 11.0 g of dry NiO and 1612 g of MgSO_4_·7H_2_O. The material balance therefore provides a quantitative basis for integrating selective ion exchange into the proposed processing route, while the overall flowsheet should be regarded as conceptual until further validated under continuous-flow and pilot-scale conditions.

Overall, the main practical advantage of the investigated system lies in the combination of high Ni(II) recovery, selectivity in a Mg-rich multicomponent sulfate matrix, operation under acidic conditions, and demonstrated performance in an actual ore-leaching solution. Of the two investigated resins, Seplite LSC 773 showed the most favorable overall performance, whereas LSC 495 remained effective under more strongly acidic conditions. These results support further development of chelating ion exchange as a selective separation stage for the integrated processing of oxidized nickel ores, with improved regeneration efficiency, extended cyclic testing, and continuous-flow validation as key priorities for future work.

## Figures and Tables

**Table 1:** Results of X-ray fluorescence (XRF) elemental analysis of the raw ore.

Element, wt.%							
O	Na	Mg	Al	P	S	Cl	Ca
52.56 ± 0.46	0.09 ± 0.01	17.16 ± 0.25	0.74 ± 0.06	0.01	0.02	0.02	0.09 ± 0.01
Cr	Mn	Fe	Co	Ni	Cu	Zn	Si
0.25 ± 0.01	0.20 ± 0.02	10.11 ± 1.03	0.05 ± 0.01	1.00 ± 0.02	0.01	0.04	17.65 ± 0.39

**Table 2:** Kinetic model parameters and statistical fitting results for Ni^2+^ adsorption onto Seplite LSC 495 and Seplite LSC 773.

Model	Parameter	LSC 495 Estimate ± SE	95% Confidence Interval	LSC 773 Estimate ± SE	95% Confidence Interval
Pseudo-first-order	q_e_,cal (mg g^−1^)	13.96 ± 0.23	13.34–14.59	14.52 ± 0.15	14.10–14.93
	k_1_ (min^−1^)	0.0547 ± 0.0061	0.0377–0.0716	0.0428 ± 0.0025	0.0358–0.0498
	R^2^	0.8584	–	0.9699	–
	RMSE (mg g^−1^)	0.3836	–	0.2491	–
Pseudo-second-order	q_e_,cal (mg g^−1^)	14.36 ± 0.16	13.92–14.80	15.00 ± 0.23	14.34–15.65
	k_2_ (g mg^−1^ min^−1^)	(9.42 ± 1.31) × 10^−3^	(5.78–13.07) × 10^−3^	(6.24 ± 0.97) × 10^−3^	(3.55–8.92) × 10^−3^
	R^2^	0.9514	–	0.9492	–
	RMSE (mg g^−1^)	0.2249	–	0.3238	–
Elovich	α (mg g^−1^ min^−1^)	(1.81 ± 19.23) × 10^9^	−5.16 × 10^10^–5.52 × 10^10^ *	(4.04 ± 37.76) × 10^6^	−1.01 × 10^8^–1.09 × 10^8^ *
	β (g mg^−1^)	2.053 ± 0.818	−0.220–4.325 *	1.550 ± 0.715	−0.435–3.535 *
	R^2^	0.6113	–	0.5402	–
	RMSE (mg g^−1^)	0.6356	–	0.9740	–
Weber–Morris	k_i_ (mg g^−1^ min^−1^/^2^)	0.0264 ± 0.0210	−0.0320–0.0848 *	0.0338 ± 0.0307	−0.0513–0.1189 *
	C (mg g^−1^)	12.833 ± 0.642	11.051–14.614	12.901 ± 0.935	10.306–15.496
	R^2^	0.2826	–	0.2330	–
Dumwald–Wagner	K_DW_ (min^−1^)	(4.84 ± 1.71) × 10^−4^	−2.50 × 10^−4^–1.22 × 10^−3^ *	0.01113 ± 0.00278	−0.00081–0.02307 *
	Intercept	−2.293 ± 0.369	−3.881–−0.706	−1.366 ± 0.475	−3.408–0.677 *
	R^2^	0.8009	–	0.8894	–
Chemisorption diffusion	K_CD_ (mg g^−1^ min^−1^/^2^)	9.82 ± 1.81	6.49–20.14 **	6.91 ± 1.57	4.24–18.65 **
	R^2^	0.8800	–	0.8295	–

Note: Values are reported as parameter estimate ± standard error (SE). Pseudo-first-order, pseudo-second-order, and Elovich parameters were obtained by nonlinear least-squares fitting of the untransformed q_t_-versus-t data. Weber–Morris, Dumwald–Wagner, and chemisorption-diffusion parameters were obtained from their corresponding linearized forms. Confidence intervals of 95% were calculated from the regression covariance matrix (nonlinear models) or from the linear regression standard errors (linearized models). * A confidence interval extending into a physically inadmissible region indicates that the corresponding parameter is poorly constrained by the present dataset and should not be interpreted as a physical range. ** For K_CD_, the 95% CI was obtained by transforming the confidence interval of the fitted reciprocal slope (K_CD_ = 1/slope) rather than by using a symmetric Wald interval. For the Dumwald–Wagner transformation, only data points for which the logarithmic term was mathematically defined were included (n = 4 for each resin); all other models used the six available kinetic data points.

**Table 3:** Comparison of representative sorption systems for Ni(II) recovery.

Sorbent	Functional Group/Mechanism	Solution and Experimental Conditions	Ni(II) Performance	Selectivity and Regeneration	Relevance to the Present Study	Reference
Seplite LSC 773	Iminodiacetate groups; chelation	Model sulfate solution; pH 3; 25 °C; contact time 5 h; solid-to-liquid ratio 1:5	Ni recovery: 99.36 ± 0.08%; adsorption capacity: 7.45 ± 0.01 mg g^−1^. In the industrial solution containing 1.68 g L^−1^ Ni, 0.33 g L^−1^ Co, and 32.00 g L^−1^ Mg, Ni recovery reached 98.59 ± 0.36%.	High selectivity toward Ni(II) in the presence of Co(II) and Mg(II). After regeneration with 20 wt.% H_2_SO_4_, adsorption capacity changed only slightly over three cycles (7.45 to 7.23 mg g^−1^), although complete desorption was not achieved.	The most promising resin for selective Ni recovery from the real Mg-rich solution; high efficiency over pH 3–6.	This work
Seplite LSC 495	Bispicolylamine groups; coordination binding	Model and industrial sulfate solutions; optimum pH 1; 25 °C; contact time 5 h; solid-to-liquid ratio 1:5	Ni recovery: 94.62 ± 0.21% from the model solution and 94.23 ± 0.21% from the industrial solution; adsorption capacity: 7.10 ± 0.02 mg g^−1^.	Most effective under strongly acidic conditions. Adsorption capacity changed from 7.09 to 7.19 mg g^−1^ over three cycles.	Confirms the suitability of the selected resins for Ni(II) recovery at low pH; potentially effective for more acidic solutions.	This work
Dowex M4195	Bispicolylamine groups; chelation	Solutions and pulps after laterite ore leaching; pH 1–4; resin-in-pulp operation	Ni and Co recovery exceeded 99% after five counter-current stages; maximum loading was 100 g Ni kg^−1^ resin.	High ability to recover Ni and Co in the presence of Fe, Al, Mg, Mn, and Cu. The system targets co-recovery of Ni and Co rather than a Ni-only product.	Supports the effectiveness of bispicolylamine resins in acidic laterite leach systems. Recovery is comparable, although process conditions differ from those of the present study.	[34]
Amberlite IRC 748	Iminodiacetate groups; complexation	Sulfate solutions containing Ni(II), Co(II), Mn(II), and Mg(II)	No universal capacity value was reported because performance depended on solution composition and pH.	Ni and Co can be separated from Mn and Mg at approximately pH 4–5. Iminodiacetate resins are sensitive to Fe(III), which competes for active sites.	Confirms selective Ni/Co recovery from multicomponent sulfate solutions; LSC 773 in the present study remained effective at a higher Mg concentration.	[34]
Lewatit MonoPlus TP 220	Bispicolylamine groups; coordination sorption	Acidic solutions, pH 2–5, including industrial acidic effluents	Exact Ni recovery was not reported in the cited study.	Equilibrium was reached after approximately 72 h. Desorption was effective with H_2_SO_4_ concentrations ≥ 5%, enabling resin reuse.	Supports the applicability of bispicolylamine resins to acidic solutions. Compared with TP 220, LSC 773 achieved high Ni recovery within 5 h.	[64]
Purolite S930	Iminodiacetate groups; chelation	Pulps of low-grade sulfide Ni–Cu ores; pH 2.7–3.0	Recovery of Cu, Ni, and Co exceeded 99%.	Highly effective for direct treatment of poorly filterable pulps; however, several metals are co-extracted, limiting Ni-specific selectivity.	Supports the use of chelating resins in complex ore systems; LSC 773 offers an advantage for selective Ni recovery from Mg-rich solutions.	[39]
Wheat-straw and rice-husk biochars	Ion exchange, electrostatic adsorption, and surface precipitation	Model aqueous Ni(II) solutions; biochars produced at 550 and 700 °C	Maximum capacities: 0.427 mmol g^−1^ for WSP700, 0.215 for WSP550, 0.173 for RH700, and 0.117 for RH550 (≈25.0, 12.6, 10.2, and 6.9 mg g^−1^, respectively).	At pH 9–10, Ni removal reached ≥98%, although part of the removal may have resulted from Ni(OH)_2_ formation.	Low-cost option for dilute aqueous solutions, but not a direct analogue of Seplite because it was not evaluated in an industrial sulfate solution with high Mg content.	[69]
Chemically modified rice husk	Oxygen-containing surface groups; ion exchange and complexation	Model aqueous Ni(II) solution; pH 4.8; 28 ± 1 °C	Ni capacities: 0.08 mmol g^−1^ for untreated husk, 0.55 mmol g^−1^ after phosphoric acid/urea treatment, and 0.75 mmol g^−1^ after phosphoric acid treatment.	Chemical modification increased adsorption capacity; data for concentrated sulfate solutions containing high Mg and Fe were not reported.	Potential low-cost material, but not a direct competitor to commercial chelating resins.	[70]
Na-mordenite	Ion exchange and surface adsorption	Model aqueous solution; initial Ni concentration 40 mg L^−1^; pH 6; 30 °C	Maximum Ni adsorption capacity: 5.371 mg g^−1^.	Effective in a single-component solution, but capacity decreases in multicomponent systems because of ion competition.	Mainly suitable for low-cost aqueous-solution treatment; less selective than chelating resins in complex sulfate media.	[71]
MnO_2_	Surface complexation through hydroxyl groups	MnSO_4_ solution containing 20 g L^−1^ Mn(II) and 80 mg L^−1^ Ni(II); pH 7; 80 °C	Ni recovery: 79.73%.	Co was simultaneously recovered; therefore, the sorbent did not exhibit high Ni selectivity.	Applicable to the purification of concentrated saline solutions, but requires near-neutral conditions and elevated temperature.	[72]

## Data Availability

The original contributions presented in this study are included in the article/Supplementary Materials. Further inquiries can be directed to the corresponding authors.

## Supplementary Materials

The following supporting information can be downloaded at https://www.mdpi.com/article/10.3390/ma19173734/s1, Figure S1: Energy diagram of elemental composition of the initial ore sample by EDS method; Table S1: Elemental composition of the initial ore sample by EDS method; Table S2: Nickel content at different analysis points of the raw laterite ore sample according to SEM–EDS measurements; Table S3: XRD peak positions of the crystalline phases identified in the original laterite ore; Figure S2: The dependence of concentration of metal ions of (a) Ni^2+^ (b) Co^2+^ and (c) Mg^2+^ in model solution on contact time (pH 495 = 1.5; T = 25 °C, S:L = 1:10); Figure S3: Performance of Ni^2+^ sorption recovery from model and industrial solutions under optimized operating conditions; Table S4: Nonlinear fitting parameters and corresponding standard errors (SE) of the adsorption isotherm models for Ni^2+^ adsorption onto Seplite LSC 495 and Seplite LSC 773; Table S5: FTIR band positions of Seplite LSC 773 and Seplite LSC 495 before and after Ni(II) adsorption; Figure S4: The effect of eluent (H_2_SO_4_) concentration on the desorption efficiency of Ni^2+^ from spent Seplite LSC 495 and Seplite LSC 773 (S:L = 1:2; t = 25 °C; τ = 2 h); Table S6: Material balance for the proposed integrated processing of nickel-bearing ore.
