# Supplementary material for: Integration of Nickel Adsorption Procedures into the Hydrometallurgical Processing of Laterite Ore: Comparative Performance of Two Chelating Ion-Exchange Resins with Insight into the Adsorption Mechanism
Source: Materials (Basel). 2026 Sep 2;19(17):3734. doi: 10.3390/ma19173734 (PMC13567613; doi:10.3390/ma19173734)
Supplement: Supplementary file 1 [file materials-19-03734-s001.zip › materials-4528532-supplementary.pdf]

Integration of Nickel Adsorption Procedures into the Hydrometallurgical Processing of Laterite Ore: Comparative Performance of Two Chelating Ion-Exchange Resins with Insight into the Adsorption Mechanism

Roza Shayakhmetova [1], Anar Mukhametzhanova [1,2,*], Almira Kuandykova [1,3], Ainur Kali [1,3,*], Petr Osipov [1] and Akmaral Rakhym [2,4]

[1] Laboratory of Rare Metals, RSE "National Center of Complex Processing of Mineral Raw Materials of the Republic of Kazakhstan", Zhandossov St., 67, Almaty 050036, Kazakhstan
[2] Department of Physical Chemistry, Catalysis and Petrochemistry, Faculty of Chemistry and Chemical Technology, Farabi University, Al-Farabi Av., 71, Almaty 050040, Kazakhstan
[3] Department of Metallurgy and Mineral Processing, Satbayev University, 22a Satpaev St., Almaty 050013, Kazakhstan
[4] Laboratory of Special Methods of Mineral Raw Materials Processing, RSE "National Center of Complex Processing of Mineral Raw Materials of the Republic of Kazakhstan", Zhandossov St., 67, Almaty 050036, Kazakhstan
* Correspondence: narike.91@gmail.com (A.M.); ainurkali04@gmail.com (A.K.)

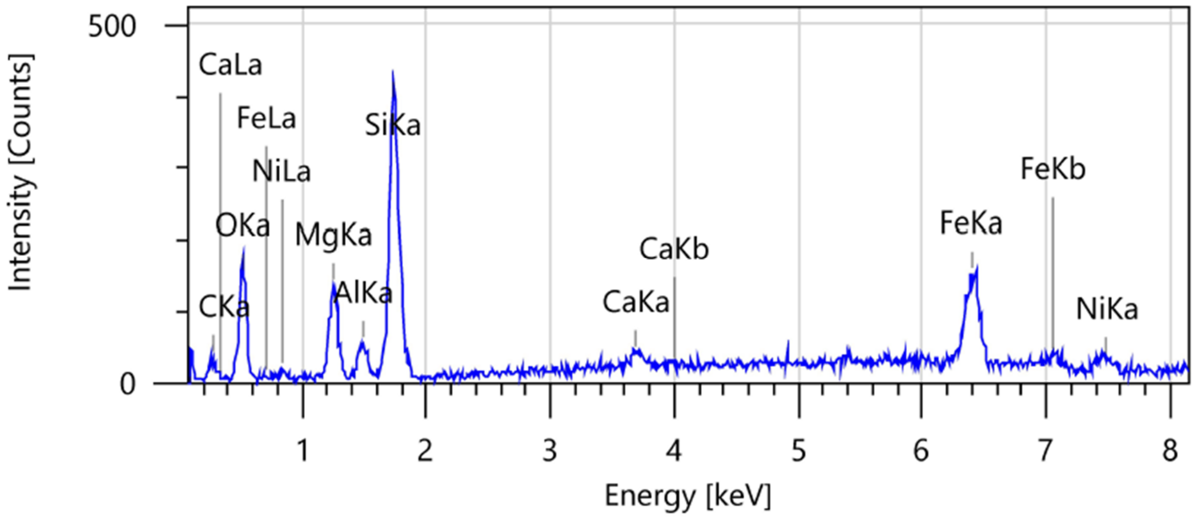

Figure S1. Energy diagram of elemental composition of the initial ore sample by EDS method.

Table S1. Elemental composition of the initial ore sample by EDS method

Element	wt.%	Standard deviation. wt.%
Ni	2.79	3.33
Fe	29.93	28.35
Si	16.57	12.41
O	37.06	17.17
Mg	6.92	5.21
C	9.24	3.43
Al	1.01	0.73
Ca	1.62	1.04
Cr	0.52	0.24
Co	0.11	n=1
Cl	2.07	n=1

Table S2. Nickel content at different analysis points of the raw laterite ore sample according to SEM–EDS measurements

Point number	Ni, wt.%	Standard deviation (±)
1	-	-
2	10.25	0.79
3	2.55	0.22
4	-	-
5	1.16	0.08
6	1.29	0.06
7	1.05	0.06
8	1.87	0.08
9	1.33	0.07
Mean	2.79	3.33

Table S3. XRD peak positions of the crystalline phases identified in the original laterite ore

Phase	Content, wt.%	2θ peak positions, °
Lizardite	15.5	12.13, 19.30, 22.83, 24.36, 31.25, 33.74, 35.95, 36.87, 39.15, 41.12, 42.03, 46.63, 49.89, 50.84, 52.61, 54.14, 54.85, 58.79, 60.30, 61.61
Goethite	2.3	20.40, 23.21, 24.50, 34.06, 36.96, 40.04, 41.99, 47.83, 51.60, 52.88, 54.23, 56.40, 60.90, 61.80, 64.18, 64.68, 72.03, 72.67
Antigorite	30.7	12.06, 19.28, 24.30, 34.16, 35.86, 38.52, 42.01, 46.21, 56.18, 60.24, 61.75, 72.10
Talc	11.9	9.49, 18.99, 19.49, 28.59, 34.58, 36.18, 40.47, 42.57, 43.67, 48.57, 53.14, 59.05, 60.72, 67.31, 71.46
Pimelite	14.9	5.85, 19.45, 26.67, 33.67, 39.67, 53.21, 60.90
Nickel silicate hydrate	11.5	9.48, 19.58, 28.68, 34.00, 35.67, 39.49, 53.04, 60.90, 71.78
Wuestite	1.8	36.00, 41.82, 60.62, 72.57, 76.36
α-Fe_2O_3	11.4	24.26, 33.33, 35.79, 39.51, 41.06, 43.71, 49.71, 54.36, 56.42, 57.72, 57.94, 62.75, 64.30, 66.37, 70.00, 72.40, 72.72, 75.83, 78.17, 79.18, 79.89

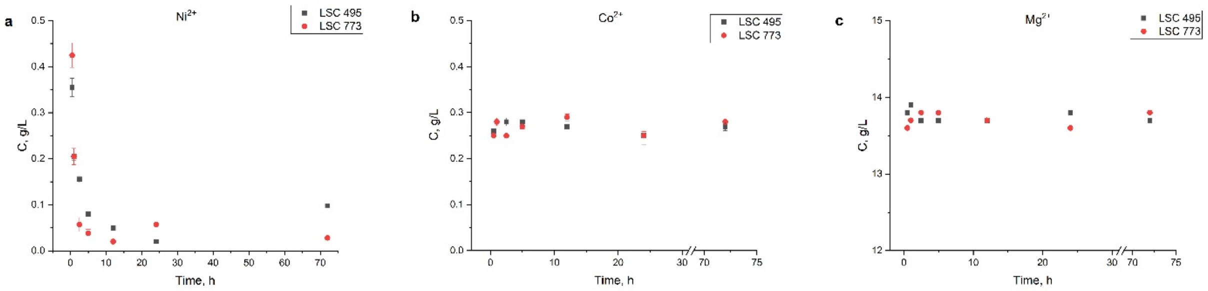

Figure S2. The dependence of concentration of metal ions of (a) Ni^{2+} (b) Co^{2+} and (c) Mg^{2+} in model solution on contact time (pH 495 = 1.5; T = 25 °C, S:L = 1:10)

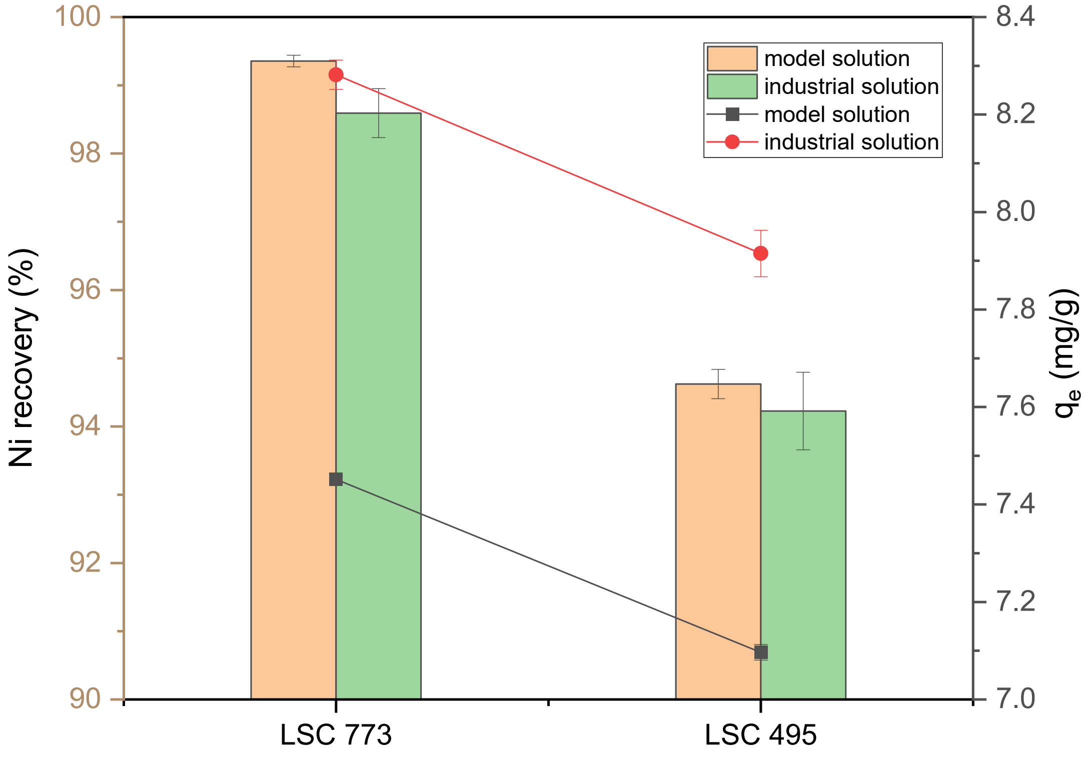

Figure S3. Performance of Ni^{2+} sorption recovery from model and industrial solutions under optimized operating conditions

Table S4. Nonlinear fitting parameters and corresponding standard errors (SE) of the adsorption isotherm models for Ni^{2+} adsorption onto Seplite LSC 495 and Seplite LSC 773.

Model	Parameter	LSC 495 Estimate	LSC 495 SE	LSC 773 Estimate	LSC 773 SE
**Freundlich**	K_F	1.020×10^{-3}	0.807×10^{-3}	3.640×10^{-6}	7.086×10^{-6}
	n	0.6622	0.0734	0.3632	0.0549
	R^2	0.9767	–	0.9673	–
	RMSE	0.0893	–	0.1158	–
**Sips**	q_m (g g⁻¹)	8.791	29.794	10.000*	51.907
	K_S	3.134×10^{-3}	8.786×10^{-3}	5.138×10^{-3}	1.217×10^{-2}
	n	1.690	0.727	3.017	1.830
	R^2	0.9770	–	0.9634	–
	RMSE	0.0888	–	0.1225	–
**Redlich–Peterson**	K_R	1.128×10^{-2}	2.984×10^{5}	1.374×10^{-2}	2.855×10^{5}
	a_R	2.377×10^{-2}	2.708×10^{7}	0.1662	2.423×10^{7}
	g	≈0	10.203	≈0	3.266
	R^2	0.9341	–	0.8230	–
	RMSE	0.1502	–	0.2694	–
**Toth**	q_m (g g⁻¹)	5.772	2.889×10^{7}	5.963	9.894×10^{7}
	K_T	1.909×10^{-3}	9.555×10^{3}	1.976×10^{-3}	3.279×10^{4}
	t	10.000*	3.513×10^{7}	9.999*	1.078×10^{8}
	R^2	0.9341	–	0.8230	–
	RMSE	0.1502	–	0.2694	–

** Parameter reached the imposed upper bound during nonlinear fitting and should therefore not be interpreted as an independently estimated physical parameter.*

Large standard errors for several Sips, Toth, and Redlich–Peterson parameters indicate poor parameter identifiability, consistent with the absence of an experimentally observed saturation plateau. These parameters are reported for comparative fitting purposes only and should not be interpreted physically.

SE values were obtained from the covariance matrix of the nonlinear least-squares regression. R^2 and RMSE are goodness-of-fit statistics and therefore no parameter SE is reported for these metrics.

The Freundlich parameters were substantially better constrained than the saturation-related parameters of the three-parameter models.

Table S5. FTIR band positions of Seplite LSC 773 and Seplite LSC 495 before and after Ni(II) adsorption

Sorbent	Band assignment	Before adsorption, cm⁻¹	After adsorption, cm⁻¹	Δν, cm⁻¹	Interpretation
Seplite LSC 773	$\nu_{as}(COO^-)$, with possible contribution from bound water	1648.4	1622.8	−25.6	Pronounced red shift consistent with the involvement of carboxylate O atoms in Ni(II) coordination.
	$\nu_s(COO^-)$	1459.1	1469.7	+10.6	Modification of the coordination and electronic environment of the carboxylate groups.
	C–N/C–O vibrations of the iminodiacetate groups	1137.8	1152.3	+14.5	Supports the participation of N- and O-containing donor groups in Ni(II) binding.
	C–O/C–N vibration in the fingerprint region	1038.0	1033.9	−4.1	Change in the local environment of the chelating functional groups.
	Skeletal and deformation vibrations of the functionalized matrix	868.3	842.5	−25.8	Pronounced rearrangement of the functionalized region after Ni(II) uptake.
	Skeletal/ring deformation vibration	773.6	769.0	−4.6	Minor but detectable change following adsorption.
Seplite LSC 495	Pyridyl-ring ν(C=N)/ν(C=C)	1622.8	1623.3	+0.5	No meaningful positional shift; changes are expressed mainly through band intensity and shape.
	Pyridyl-ring vibration	1593.4	1594.8	+1.4	Shift is close to the practical peak-picking uncertainty.
	Pyridyl-ring ν(C=C)/C–H bending	1508.3	1507.8	−0.5	No significant positional shift.
	Pyridyl-ring/C–N vibration	1469.7	1470.9	+1.2	Small change in the electronic environment of the pyridyl donor groups.

	C–N stretching of the bis-picolylamine ligand	1152.3	1151.3	−1.0	No substantial shift; relative intensity changes may be more informative.
	C–N/pyridyl-ring vibration	1106.9	1097.8	−9.1	Distinct shift consistent with perturbation of the pyridyl and amine donor environment during Ni(II) coordination.
	Pyridyl-ring breathing vibration	1033.9	1035.1	+1.2	Minor positional change accompanied by a change in relative intensity.
	Out-of-plane C–H deformation of the pyridine ring	842.5	849.2	+6.7	Indicates perturbation of the pyridyl-ring environment after Ni(II) uptake.
	Pyridine-ring out-of-plane deformation	769.0	768.5	−0.5	No meaningful positional shift.

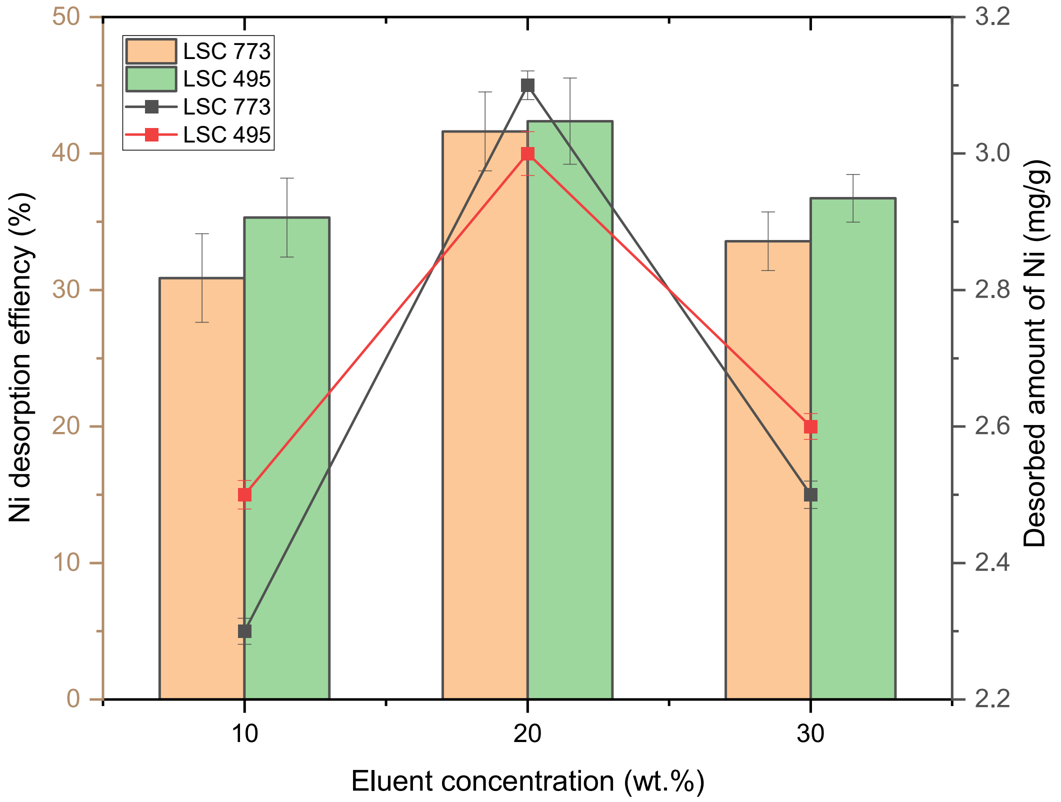

Figure S4. The effect of eluent (H_2SO_4) concentration on the desorption efficiency of Ni^{2+} from spent Seplite LSC 495 and Seplite LSC 773 (S:L = 1:2; t = 25 °C; τ = 2 h)

Table S6. Material balance for the proposed integrated processing of nickel-bearing ore

Process stage	Input				Output			
	Stream/material	Amount, g	Ni, g	Mg, g	Stream/material	Amount, g	Ni, g	Mg, g
Ore preparation	Initial ore	1000	10	172	Ground ore	1000	10	172
Leaching	Ground ore	1000	10	172	Pregnant leach solution	4607	8.64	159.8
	Concentrated H_2SO_4	977			Wet SiO_2 cake	1300	1.56	12.2
	Wash water	1327	1.5	0.2	–	–		
	Water	2603			–	–		
	**Total:**	5907			**Total:**	5907		
Washing and drying	Wet SiO_2 cake	1300	1.56	12.2	Silica-rich cake (dry)	660	0.06	0.2
	Wet SiO_2 cake	1300	1.56	12.2	Wash water	1327	1.5	12
	H_2O	1300	–	–	Vapor	613		
	**Total:**	2600			**Total:**	2600		
Hydrolytic Fe precipitation	Pregnant leach solution	4607	8.64	159.8	Solution after precipitation	4400	8.64	159.8
	10% NaOH solution, 1.108 g cm^{-3}	510			Wet Fe cake	682		
	Praestol 2500	5			Losses	40		
	**Total:**	5122			**Total:**	5122		
Washing and drying	Wet Fe cake	682			Dry Fe cake	191		
	H_2O for two washing stages	2050			Vapor	491		
	–	–			Wash solutions from three washing stages, 1:1	2050		
	**Total:**	2732			**Total:**	2732		
Ni sorption	Solution after precipitation	4400 p=1.3 г\см3	8.64	159.8	Sorbate	4386	–	159.8
	LSC 773, 40% moisture	37	–	–	Ni-loaded sorbent	47	8.64	–
	–	–	–	–	Sorbate losses	4	–	–
	**Total:**	4437	–	–	**Total:**	4437	–	–
Co precipitation	Sorbate	4386	–		$MgSO_4$ solution	4445	–	175.5
	MgO	26	–		Wet Co-containing product	5	–	
	H_2O_2	38	–					
	**Total:**	4450			**Total:**	4450		
Drying and calcination	Wet Co-containing product	5			Dehydrated Co-containing product	2		
	–	–			Vapor	3		

Process stage	Input				Output			
	**Stream/material**	**Amount, g**	**Ni, g**	**Mg, g**	**Stream/material**	**Amount, g**	**Ni, g**	**Mg, g**
	**Total:**	5			**Total:**	5		
Evaporation	p-p MgSO4	4450		175.5	$MgSO_4$ solution	3150		175.5
		–			Vapor	1300		
	**Total:**	4450			**Total:**	4450		
Crystallization/filtration	$MgSO_4$ solution	3150	–	175.5	$MgSO_4 \cdot 7H_2O$	1612		159.0
	–	–	–		$MgSO_4$ mother liquor	1538		16.5
	**Total:**	3150			**Total:**	3150		
Drying	$MgSO_4 \cdot 7H_2O$	1612	–	159.0	$MgSO_4$	788		159.0
	–	–			Vapor	824		
	**Total:**	1612			**Total:**	1612		
Desorption	Ni-loaded sorbent	47	8.64		$NiSO_4$ solution	1080	8.64	
	20% H_2SO_4 solution; 1.139 g cm^{-3}	1070			LSC 773 sorbent for regeneration	37		
	Ni-free H_2SO_4 solution	360			Ni-free H_2SO_4 solution	360		
	**Total:**	1477			**Total:**	1477		
Ni precipitation	$NiSO_4$ solution	1080	8.64		$NiCO_3$, 60% moisture	43.75	8.64	
	10% Na_2CO_3 solution	156			Na_2SO_4 solution	1192.25		
	**Total:**	1236			**Total:**	1236		
Drying at 105 °C and calcination at 350 °C	$NiCO_3$, 60% moisture	43.75	8.64		Dry NiO	11.0	8.64	
	–	–			Vapor	26.25		
					CO_2	6.5		
	**Total:**	43.75			**Total:**	43.75		
